# Transcriptional profiling of zebrafish intestines identifies macrophages as host cells for human norovirus infection

**DOI:** 10.1080/19490976.2024.2431167

**Published:** 2024-11-25

**Authors:** Emma Roux, Reegan J. Willms, Jana Van Dycke, Álvaro Cortes Calabuig, Lore Van Espen, Geert Schoofs, Jelle Matthijnssens, Johan Neyts, Peter de Witte, Edan Foley, Joana Rocha-Pereira

**Affiliations:** a Department of Microbiology, Immunology and Transplantation, Rega Institute, Virus-Host Interactions & Therapeutic Approaches (VITA) Research Group, KU Leuven, Leuven, Belgium; b Department of Medical Microbiology and Immunology, Faculty of Medicine and Dentistry, University of Alberta, Edmonton, Alberta, Canada; c Department of Human Genetics, Genomics Core Leuven, KU Leuven, Leuven, Belgium; d Department of Microbiology, Immunology and Transplantation, Rega Institute, Laboratory of Clinical and Epidemiological Virology, KU Leuven, Leuven, Belgium; e Department of Microbiology, Immunology and Transplantation, Rega Institute, Molecular Structural and Translational Virology Research Group, KU Leuven, Leuven, Belgium; f Department of Microbiology, Immunology and Transplantation, Rega Institute, Virology, Antiviral Drug & Vaccine Research Group, KU Leuven, Leuven, Belgium; g Department of Pharmaceutical and Pharmacological Sciences, Laboratory for Molecular Biodiscovery, KU Leuven, Leuven, Belgium

**Keywords:** Human norovirus, cellular tropism, macrophages, intestinal epithelium, host cell identification, host-virus interaction, zebrafish larval model

## Abstract

Human noroviruses (HuNoVs) are a major cause of diarrheal disease, yet critical aspects of their biology, including cellular tropism, remain unclear. Although research has traditionally focused on the intestinal epithelium, the hypothesis that HuNoV infects macrophages has been recurrently discussed and is investigated here using a zebrafish larval model. Through single-cell RNA sequencing of dissected zebrafish intestines, we unbiasedly identified macrophages as host cells for HuNoV replication, with all three open reading frames mapped to individual macrophages. Notably, HuNoV preferentially infects actively phagocytosing inflammatory macrophages. HuNoV capsid proteins and double-stranded RNA colocalized within intestinal macrophages of infected zebrafish larvae, and the negative-strand RNA intermediate was detected within FACS-sorted macrophages. Flow cytometry confirmed viral replication within these macrophages, constituting approximately 23% of HuNoV’s host cells. Identifying macrophages as host cells prompts a reevaluation of their role in HuNoV pathogenesis, offering new directions for understanding and controlling this infection.

## Introduction

The development of antivirals and vaccines against human norovirus (HuNoV) is a recognized public health need as this gastroenteric virus causes high mortality in young children^1^ and significant morbidity in chronically infected patients, affecting up to 18% of transplant patients.^2,3^ Traditional research obstacles, including the lack of *in vitro* and *in vivo* models of HuNoV infection, have been overcome by the human intestinal enteroid (HIE) model^4^ and our *in house* developed zebrafish larvae model.^5^ These models, while not being the most common, provide robust and reproducible platforms, expanding our knowledge of HuNoV biology, namely regarding its cellular tropism and interactions with the host. Unraveling unknown details on HuNoV biology will boost the development of specific therapeutics and increase our understanding of gastroenteric viruses, their replication, and disease-inducing mechanisms.

The discovery that the mouse norovirus (MNV) targets macrophages and dendritic cells in the gut-associated lymphoid tissue, brought a paradigm shift on norovirus tropism, suggesting broader susceptibility beyond the intestinal epithelium.^6,7^ However, infection of immune cells was considered improbable for HuNoV, especially since enterocytes were identified as the sole HuNoV permissive cell type in HIE cultures,^4^ in accordance with an earlier study using biopsies of immunocompromised patients.^8^ Subsequently, evidence of infection of enteroendocrine cells was revealed through analysis of intestinal biopsies from HuNoV-infected immunocompromised patients.^9^

Yet, macrophages and dendritic cells in the lamina propria of these biopsies were also found to colocalize with HuNoV capsid protein.^8,9^ Additionally, HuNoV was effectively cultured in B cells *in vitro* and research involving chimpanzees and miniature piglets pinpointed macrophages, dendritic cells, and lymphocytes as host cells for HuNoV.^10–13^ Although these results hint that murine and human noroviruses share a dual cell tropism for both intestinal epithelial and immune cells, it is not consensual to which extent and under which circumstances HuNoV can be present and replicate in immune cells.

We have established a robust replication model for HuNoV using zebrafish larvae.^5,14^ Zebrafish are widely used as research models and have gathered interest in virology in the past decade with the development of a model for e.g., herpes simplex 1, chikungunya, and influenza A virus.^15–17^ Their high degree of genetic and physiological similarity is of interest, particularly the resemblance to the human immune system and intestinal architecture.^18–20^ The use of this zebrafish model to study the *in vivo* cellular tropism of HuNoV specifically has multiple advantages. Their optical transparency and the availability of transgenic lines expressing fluorescently labeled cells make zebrafish larvae a powerful next-generation infection model that allows scientists to illuminate unknown details of virus-host interactions and viral pathogenesis.^21^

Using a zebrafish larval model, this study investigated the role of macrophages and neutrophils, the main players in the innate immune response, in HuNoV infection and clearance. We evaluated whether the tropism of MNV for immune cells is shared by HuNoV by a combination of immunofluorescence, flow cytometry, strand-specific RT-PCR, and single-cell RNA-sequencing (scRNA-seq). In summary, the research conclusively establishes that macrophages are permissive target cells for HuNoV in zebrafish larvae, while neutrophils play a lesser role in HuNoV infection.

## Materials and methods

### Ethics statement

All zebrafish experiments were approved and performed according to the rules and regulations of the Ethical Committee of KU Leuven (P066/2022), in compliance with the European Union regulations concerning the welfare of laboratory animals as declared in Directive 2010/63/EU. Human stool samples positive for HuNoV were obtained from the University Hospital of Leuven (UZ Leuven, Belgium) according to the rules and regulations of the Ethical Committee of KU Leuven (G-2021–4376) and the UZ Leuven (S63536).

### Zebrafish husbandry and transgenic lines

Adult zebrafish (wildtype AB, Tg(mpx:GFP)^i114^ (further Tg(mpx:GFP)), Tg(mpeg:mCherry-F)^ump2Tg^ (further Tg(mpeg:mCherry-F)), Tg(fms:Gal4VP16^i186^/UAS:E1b:nfsB:mCherry^i149^) x Tg(mpx:GFP)^i114^ (further Tg(fms:Gal4/UAS:nfsB:mCherry/mpx:GFP)), Tg(mpeg1:Gal4FF^gl25^/UAS:E1b:nfsB:mCherry^i149^) (further Tg(mpeg1:Gal4/UAS:nfsBmCherry)), and irf8^−/−^mutants, derived from heterozygous incrosses of irf8^±^ fish; were maintained in the aquatic facility of the KU Leuven (temperature of 28°C and 14/10 h light/dark cycle). Fertilized eggs were collected from adults placed in mating cages and kept in Petri dishes containing Danieau’s solution (1.5 mM HEPES, 17.4 mM NaCl, 0.21 mM KCl, 0.12 mM MgSO_4_, and 0.18 mM Ca(NO_3_)_2_ and 0.6 μM methylene blue) at 28°C until the start of experiments.

### Processing of HuNoV-positive stool samples

A human stool sample, positive for HuNoV (GII.4 Sydney [P4] 2012), was obtained from the University Hospital of Leuven (Belgium). An aliquot of 100 mg of the stool sample was re-suspended in 1 mL of sterile Gibco Dulbecco’s phosphate-buffered saline (DPBS, Thermo Fisher Scientific, Waltham, Massachusetts, USA), thoroughly vortexed and centrifuged (5 min 10,000 *g*), the supernatant was harvested, aliquoted and stored at −80°C. This virus suspension was used for injections in the zebrafish larvae.

### HuNoV infection of zebrafish larvae

Zebrafish larvae were anesthetized using tricaine (Sigma-Aldrich, Saint Louis, Missouri, USA) and positioned as described previously,^5,14,22^ in short; zebrafish larvae of 3 dpf were anesthetized and transferred to an agarose mold to position them on their dorsal side with the yolk facing upward. In every experiment, the injection needle was calibrated to ensure the precision of the injection volume. Microinjection was performed using an M3301R Manual Micromanipulator (WPI, Friedberg, Germany) and a FemtoJet 4i pressure microinjector (Eppendorf, Hamburg, Germany). The zebrafish larvae received an injection of 3 nL virus in the yolk. After injection, zebrafish larvae were transferred to 6-well plates with Danieau’s solution and further maintained in an incubator with a 14/10 h light/dark cycle at 32°C.

### RNA extraction and viral RNA quantification

Each day post-injection, 10 zebrafish larvae were collected into 2 mL tubes containing 1.4 mm ceramic beads (Omni International, Kennesaw, Georgia, USA) and stored at 80°C until further processing. First, 350 µL of Trizol™ Reagent (Thermo Fisher Scientific, Waltham, Massachusetts, USA) was added to the microtubes containing the harvested zebrafish larvae. Samples were then homogenized for 10 s at 6,300 rpm (Bertin Technologies, Montigny-le-Bretonneux, France). Next, the homogenate was cleared by centrifugation (2 min, 100,000 *g*), and the supernatant was transferred to a sterile Eppendorf tube. An equal volume of absolute (98%-100%) ethanol was added to the homogenate followed by the extraction of the viral RNA using the Direct-zol™ RNA Miniprep kit (Zymo Research, Irvine, California, USA) according to the manufacturer’s protocol. The extracted RNA was stored at −80°C for storage until quantification by RT-qPCR. The detection of viral RNA was performed via a one-step RT-qPCR using the iTaq Universal Probes One-Step Kit (Bio-Rad, Hercules, California, USA). Primers and probes are listed in Table 1. Cycling conditions were: reverse transcription at 50°C for 10 min, initial denaturation at 95°C for 3 min, followed by 40 cycles of amplification (95°C for 15 s, 60°C for 30 s) (Quantstudio™ 5 instrument (Thermo Fisher Scientific, Waltham, Massachusetts, USA). For absolute quantification, standard curves were generated using 10-fold dilutions of template DNA of known concentration.

**Table 1.** Primers and probes used for RT-qPCR quantification of viral RNA.

Primer	Sequence 5'–3'	Reference
QNIF2	ATGTTCAGRTGGATGAGRTTCTCWGA	23
COG2R	TCGACGCCATCTTCATTCACA	
Probe	Sequence 5'–3'	Reference
Ring2	FAM-TGGGAGGGCGATCGCAATCT- TAMRA	24

### Fluorescence-activated cell sorting of macrophages

The preparation of a single-cell suspension of zebrafish larvae of the transgenic line Tg(mpeg:mCherry-F) for subsequent fluorescent sorting was based on a published protocol.^25^ In short, zebrafish larvae of 5 dpf were first anesthetized using tricaine (Sigma-Aldrich, Saint Louis, Missouri, USA) followed by a 15 min washing step in calcium-free Ringer solution (Thermo Fisher Scientific, Waltham, Massachusetts, USA). Next, zebrafish larvae were dissociated in 0.25% trypsin-1 mM EDTA solution (Gibco, Waltham, Massachusetts, USA) which was pre-heated at 28°C while pipetting up and down for 60 min. Afterwards CaCl_2_ and fetal calf serum (Gibco, Waltham, Massachusetts, USA) were added to a final concentration of 1 mM and 10%, respectively. The cell suspension was washed twice using centrifugation for 3 min at 800 *g*. Next, the cell suspension was incubated using a Zombie UV viability dye (Biolegend, San Diego, California, USA) followed by two additional washing steps. The final cell suspension was filtered through a 35 µm nylon mesh cell strainer (Corning, New York, USA) and subjected to FACS-sorting using the BD influx cell sorter linked to BD FACS software (BD Biosciences, New Jersey, USA).

### Strand specific detection of HuNoV RNA via PCR

Specific amplification of positive- and negative-sense HuNoV RNA was performed based on an earlier described protocol.^26^ For first-strand synthesis, 1 µL of RNA extract (extract of whole zebrafish larvae) or 10 µL of RNA extract (extract of FACS-sorted macrophages) was mixed with 1 µL 10 mM DNTP mix (Thermo Fisher Scientific, Waltham, Massachusetts, USA), 1 µL of 10 µM primer solution (HuNoV-F1-TAG for negative-strand assay, HuNoV-R1-TAG for positive-strand assay) and diluted with DEPC-treated water to 13 µL. Primer sequences are listed in Table 2. Samples were heated at 65°C for 5 min and incubated on ice for at least 1 min. To each sample, 4 µL of 5X first strand buffer (Thermo Fisher Scientific, Waltham, Massachusetts, USA), 1 µL of 0.1 M DTT, 1 µL of RNasin Plus (Promega, Madison, Wisconsin, USA), and 1 µL of Superscript IV reverse transcriptase (Thermo Fisher Scientific, Waltham, Massachusetts, USA) was added. Samples were incubated at 55°C for 10 min and 80°C for 10 min. Next, 40 units of Exonuclease I (New England Biolabs, Ipswich, Massachusetts, USA) and 4 µL of Shrimp Alkaline Phosphatase (New England Biolabs, Ipswich, Massachusetts, USA) were added. Samples were incubated for 15 min at 37°C followed by an inactivation for 15 min at 80°C. DNA was purified using a Qiagen PCR purification kit according to the manufacturer’s instructions (Qiagen, Hilden, Germany). Five µL of the eluate was further used in a first PCR reaction using the GoTaq Hotstart MasterMix (Promega, Madison, Wisconsin, USA) and primer set HuNoV-F1/TAG for the positive-strand assay and primer set HuNoV-R1/TAG for the negative-strand assay. The PCR reaction was conducted at the following temperature scheme: 2 min at 95°C, 40X (30 s at 95°C + 30 s at 530°C +1 min at 72°C), and 5 min at 72°C. DNA was purified using a Qiagen PCR purification kit according to the manufacturer’s instructions (Qiagen, Hilden, Germany). One µL of the eluate was further used in a second PCR reaction using the GoTaq Hotstart MasterMix (Promega, Madison, Wisconsin, USA) and primers HuNoV-F2 and HuNoV-R2. Amplified products were separated on a 2% agarose gel (Expected fragment length was 376 bp). Extracts of both whole fish (uninfected and HuNoV-infected) and uninfected FACS-sorted macrophages were used as controls.

**Table 2.** Primers used for RT-PCR detection of positive- and negative-sense viral RNA.

Primer	Sequence 5'–3'
HuNoV-F1	GCACAGACATAAAATTGGACCCAGAG
HuNoV- F1-TAG	CGGTCATGGTGGCGAATAAGCACAGACATAAAATTGGACCCAGAG
HuNoV-R1	CATTCTGGCCAAATGGGATAGATAGG
HuNoV-R1-TAG	CGGTCATGGTGGCGAATAACATTCTGGCCAAATGGGATAGATAGG
TAG	CGGTCATGGTGGCGAATAA
HuNoV-F2	CTGGAGCAGAGTTCAATACTCAGGC
HuNoV-R2	AACCTCATTGTTGACCTCTGGGAC

### Flow cytometry

A flow cytometry protocol was used and adapted from a published protocol.^27^ Zebrafish larvae of 5 dpf were first anesthetized using tricaine (Sigma-Aldrich, Saint Louis, Missouri, USA) before dissecting the larvae using carbon steel surgical blades (SwannMorton, Sheffield, United Kingdom). Next, zebrafish lysates were digested by the use of a digestion cocktail consisting of 200 μL enzymatic mix (10 mg/mL collagenase (Sigma-Aldrich, Saint Louis, Missouri), 5 mg/mL hyaluronidase (Sigma-Aldrich, Saint

Louis, Missouri, USA), 20 mg/mL proteinase K (Invitrogen, Waltham, Massachusetts, USA)) in 3 mL of 0.25% trypsin-1 mM EDTA solution (Gibco, Waltham, Massachusetts, USA). Zebrafish lysates were incubated in the digestion cocktail at room temperature (RT) under constant shaking for 20 min. Zebrafish lysates were then filtered through a 40 µm nylon mesh (Corning, New York, USA) pre-moistened with L-15 medium (Gibco, Waltham, Massachusetts, USA) supplemented with 10% fetal bovine serum (Gibco, Waltham, Massachusetts, USA). Dissociation was stopped by adding 20 mL of L15 medium to the solution, followed by centrifugation for 5 min at 800 *g* at 4°C. Supernatant was removed and 20 mL of DPBS was added to the cell pellet followed by centrifugation for 5 min at 800 *g* at 4°C. Cells were resuspended in 1 mL DPBS and filtered through a 35 µm nylon mesh cell strainer (Corning, New York, USA). The cell concentration and viability were measured using a LUNA II automated cell counter (Logos Biosystems, Anyang, Kyonggi-do, South Korea). Hereafter, cells were stained with fixable viability stain 780 (BD Biosciences, New Jersey, USA) in a 1:1,000 dilution for 15 min in the dark at RT. After two subsequent washing steps in staining buffer, cells were fixated and permeabilized using the BD Cytofix/Cytoperm™ Fixation/Permeabilization Solution Kit (BD Biosciences, New Jersey, USA) for 20 minutes at 4°C. After this step, cells were washed two times in Cytoperm™ buffer followed by blocking in Cytoperm™ buffer containing 10% (v/v) sheep serum (SigmaAldrich, Saint Louis, Missouri, USA) for 30 min at 4°C. Primary antibodies (Table 3) were diluted in Cytoperm™ buffer-10% (v/v) sheep serum (Sigma-Aldrich, Saint Louis, Missouri, USA) and incubated for 30 min at 4°C followed by two washing steps of 5 min at RT in perm wash buffer. Following that, the cells were incubated for 20 min at 4°C with secondary antibodies (Table 3), once again followed by two washing steps as described previously. Finally, cells were resuspended in 1 mL of staining buffer before readout using BD LSRFortessa X20 (BD Biosciences, New Jersey, USA). Data was analyzed using FlowJo software 10.8.0 (BD Biosciences, New Jersey, USA).

**Table 3.** Primary and secondary antibodies used for flow cytometry and whole mount immunohistochemistry.

Antibody	Host	Dilution	Supplier
1°anti-mCherry IgG	Rabbit	1:500 (IF) 1:1,000 (FCM)	ChromoTek (Planegg-Martinsried, Germany)
1° anti-GFP IgG	Rabbit	1:500 (IF)	ChromoTek (Planegg-Martinsried, Germany)
1° anti-VP1 IgG	Mouse	1:100 (IF) 1:200 (FCM)	Provided by Dr. Peter Sander; *R*- Biopharm (Darmstadt, Germany)
1°anti-dsRNA IgG J2	Mouse	1:200 (IF) 1:400 (FCM)	Jena Bioscience (Jena, Germany)
AF647-labeled anti-dsRNA IgG	Mouse	1:100 (IF)	Jena Bioscience (Jena, Germany), Invitrogen (Waltham, Massachusetts, USA)
2° anti-mouse IgG cross-absorbed AF488	Goat	1:1,000	Invitrogen (Waltham, Massachusetts, USA)
2° anti-rabbit IgG cross-absorbed AF488	Goat	1:1,000	Invitrogen (Waltham, Massachusetts, USA)
2° anti-mouse IgG cross-absorbed AF594	Goat	1:1,000	Invitrogen (Waltham, Massachusetts, USA)
2° anti-rabbit IgG cross-absorbed AF594	Goat	1:1,000	Invitrogen (Waltham, Massachusetts, USA)

### Whole mount immunohistochemistry and imaging

Whole mount immunofluorescence stainings were performed based on an earlier published protocol.^28^ In short, 24 hpf zebrafish embryos were treated with 75 µM 1-phenyl 2-thiourea (PTU) (Tokyo Chemical Industry Co., Tokyo, Japan). At 5 dpf anaesthetized zebrafish larvae were fixated for 2 h in 4% paraformaldehyde in PBS.

After fixation, larvae were rinsed with autoclaved Milli-Q water for 30 min while shaking. Next, larvae were immersed in cold 100% acetone for 20 min at −20°C. Thereafter, larvae were washed thrice with 0.01% Tween-20 in Gibco™ Hanks’ Balanced Salt Solution (HBSS) (Thermo Fisher Scientific, Waltham, Massachusetts, USA) and permeabilised in 1.5 mg/mL collagenase (Sigma-Aldrich, Saint-Louis, Missouri, USA) in HBSS containing 0.01% Tween +5 mM CaCl_2_ for 2 h at room temperature (RT). Next, larvae were blocked with 10% sheep serum (Sigma-Aldrich, Saint Louis, Missouri) in PBSDT (PBS +0.1% Triton + 1% DMSO)) for 2 h at RT. After, primary antibody (Table 3) in blocking solution was added and kept either overnight or for 36 h (anti-VP1 antibody) at 4°C. Following antibody incubation, the larvae were washed 6 times for 15 min and 3 times for 30 min in PBSDT and blocked for 2 h in blocking solution. Next, secondary antibody (Table 3) in blocking solution was added and incubated overnight at 4°C. In case of an additional incubation with a directly-labeled antibody (Supplementary Figure S4), this was followed by washing 6 times for 15 min and 3 times for 30 min in PBSDT and subsequent blocking in 10% sheep serum for 2 h. An overnight incubation with the directly-labeled antibody in blocking solution was performed afterward. Next, larvae were washed twice for 15 min with PBSDT followed by a counterstaining with 2 µg/mL Hoechst 33,352 (Thermo Fisher Scientific, Waltham, Massachusetts, USA) in PBT (PBS +0.1% Tween-20) for 30 min. Finally, zebrafish larvae were washed twice for 15 min and four times for 30 min in PBT. After staining, zebrafish larvae were progressively stored in 80% (v/v) glycerol in PBT and kept at 4°C until imaging.

**Figure 1.**
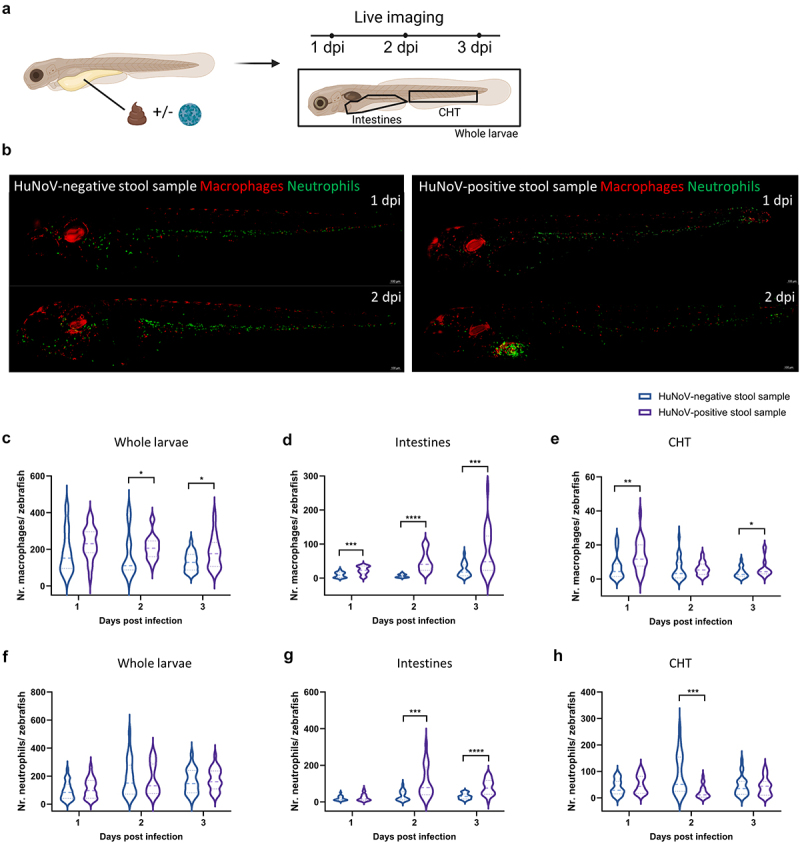
Upon HuNoV infection macrophages expand and are attracted to the intestines. (a) A schematic overview illustrating the experimental setup where zebrafish larvae were injected with either a HuNoV-negative or -positive stool sample, followed by live imaging at 1, 2, and 3 dpi. (b) Representative fluorescent images (10X magnification) of zebrafish larvae from the transgenic line Tg(fms:Gal4/UAS:nfsB:mCherry/mpx:GFP) injected with HuNoV-negative or -positive stool samples. The images show the recruitment of macrophages (red) and neutrophils (green) to the intestinal region where HuNoV infection occurs. Images were deconvoluted using leica LAS X imaging software. (c-e) quantification of macrophages in (c) whole larvae, (d) intestines, and (e) the caudal hematopoietic tissue (CHT) using ImageJ, in zebrafish injected with either a HuNoV-negative or positive stool sample. (f-h) quantification of neutrophils in (f) whole larvae, (g) intestines, and (h) the CHT using ImageJ, in zebrafish injected with either a HuNoVnegative or -positive stool sample. For panels (c-h), data from 22-24 larvae per condition are shown as violin plots with medians, with outliers removed (ROUT, Q = 1%). Statistical analysis was performed using the Mann-Whitney test, with significant differences marked by asterisks: *p < 0.05, **p < 0.01, ***p < 0.001, ****p < 0.0001. In (c), macrophage numbers increased by 1.26-fold (*p* = 0.0135) at 2 dpi and 1.38-fold (*p* = 0.0431) at 3 dpi. In (d), macrophages increased by 2.36-fold (*p* = 0.003) at 1 dpi, 7.65-fold (*p* < 0.0001) at 2 dpi, and 3.29-fold (*p* = 0.0005) at 3 dpi. In (r), macrophage numbers in the CHT increased by 2.00-fold (*p* = 0.0038) at 1 dpi and 1.69-fold (*p* = 0.044) at 3 dpi. In (g), neutrophil numbers in the intestines increased by 3.21-fold (*p* = 0.0002) at 2 dpi and 2.60-fold (*p* < 0.0001) at 3 dpi, while (h) shows a 3.91 fold decrease (*p* = 0.0002) in neutrophils in the CHT at 2 dpi. HuNoV = human norovirus, dpi = days post-infection, CHT = caudal hematopoietic tissue.

For Figures 1, 7(b,d) and Supplementary Figure S12, images were taken on a Leica DMi8 inverted fluorescent microscope (Leica Microsystems, Wetzlar, Germany) and processed with the associated Leica Application Suite X (LAS X) software. Larvae were imaged at a 10X magnification as z-stacks, processed by the 3D-Deconvolution software of LAS X, and presented as maximum projections. For Figure 4, Supplementary Figure S2-S6, and 10–11 images were taken on a Andor Dragonfly 200 series High Speed confocal platform system (Oxford instruments, Abingdon, UK) at a 25× magnification connected to a Leica DMi8 (Leica Microsystems, Wetzlar, Germany). Image processing and 3D colocalization analysis were done with the Imaris analysis software (Oxford instruments, Abingdon, UK) and images are presented as maximum projections.

For Figures 1 and 7(b,d), quantification of leukocytes using ImageJ software was performed as previously described.^29^ In short, the total fluorescence of thresholded binary images was measured alongside the leukocyte size for each experimental condition (macrophages/neutrophils, uninfected/HuNoV-infected, and 1/2/3 dpi). Subsequently the total fluorescence was divided by the average leukocyte size obtained by measuring 5 cells per embryo with a total of 5 embryos per experimental condition.

### Generation of single-cell suspension from dissected intestines for single-cell RNA-seq

The generation of a single-cell suspension of dissected intestines for subsequent single-cell RNA-seq analysis was performed as described before.^30^ To summarize, five larvae were euthanized per time using an overdose of tricaine (Sigma-Aldrich, Saint Louis, Missouri, USA), after which intestines were immediately dissected using sterilized #5SF Super Fine Tweezers (Dumont, Montignez, Switzerland) which were pre-incubated in 1% bovine serum albumin (BSA) (Sigma-Aldrich, Saint Louis, Missouri, USA) and placed in 200 µL of PBS on ice. In total, 30 intestines were dissected per replicate and 2 replicates per condition were included. Immediately after dissections, intestines were placed in 1.5 mL Eppendorf tubes containing 200 µL of dissociation cocktail containing 1 mg/mL collagenase (Sigma-Aldrich, Saint-Louis, Missouri, USA), 40 µg/mL proteinase K (Thermo Fisher Scientific, Waltham, Massachusetts, USA), and 0.25% trypsin (Thermo Fisher Scientific, Waltham, Massachusetts, USA) in PBS using 1% BSA pre-soaked tips. Dissociation was performed for 40 min at 37°C while pipetting up and down every 10 min for optimal digestion. To stop digestion, 10% BSA in PBS was added to the dissociation cocktail to halt digestion and cells were spun for 15 min at 300 *g* at 4°C to pellet the cells. The cells were resuspended in 200 µL of 0.04% BSA in PBS and filtered through a 40 µm filter (pluriSelect Life Science, Leipzig, Germany) and spun for 1 min at 300 *g* at 4°C. Live cells were selected via the OptiPrep Density Gradient Medium (Sigma-Aldrich, Saint Louis, Missouri, USA) according to the manufacturer’s protocol. The cell concentration and viability were measured using a LUNA II automated cell counter (Logos Biosystems, Anyang, Kyonggi-do, South Korea). The single-cell suspensions were immediately subjected to the 10X Genomics Chromium Controller with Chromium Single Cell 3′ Library & Gel Bead Kit v3.1. Libraries were constructed according to 10X Genomics Chromium Single Cell 3’ Library & Gel Bead Kit v3 protocol. Paired-end sequencing was performed by the Genomics Core Leuven on the Illumina Novaseq 6000 system.

### FACS-sorting macrophages for single-cell RNA-seq

First, 500 zebrafish of the line Tg(mpeg:mCherry-F) were sedated using tricaine (Sigma-Aldrich, Saint Louis, Missouri, USA) followed by rinsing in calcium-free KrebsRinger solution (Thermo Fisher Scientific, Waltham, Massachusetts, USA) for 5 min. Next, zebrafish larvae were digested using a pre-heated (28°C) solution of 0.25% trypsin-1 mM EDTA solution (Gibco, Waltham, Massachusetts, USA) supplemented with 2 mg/mL collagenase (Sigma-Aldrich, Saint-Louis, Missouri, USA) for 30 min while pipetting up and down every 5 min. The generated cell suspension was filtered through a 70 µm cell strainer (Corning, New York, USA) and dissociation was stopped by the addition of CaCl_2_ (1 mM) (Sigma-Aldrich, Saint Louis, Missouri, USA) and 10% fetal bovine serum (Gibco, Waltham, Massachusetts, USA). The cell pellet was collected after centrifugation at 500 *g* for 5 min and rinsed using PBS containing 10% fetal bovine serum (Gibco, Waltham, Massachusetts, USA). Cells were stained using a live-death Zombie UV fixable viability stain (Biolegend, San Diego, California, USA) for 20 min followed by a washing step using PBS (10% fetal bovine serum). After centrifugation at 500 *g* for 5 min, the cell pellet was resuspended in PBS (10% fetal bovine serum) and filtered through a 35 µm nylon mesh cell strainer (Corning, New York, USA). Cell sorting was performed at 4°C using the BD Influx™ cell sorter. Cells of interest were sorted in a collection tube containing 100 µL of fetal bovine serum (Gibco, Waltham, Massachusetts, USA) that was pre-coated with PBS (20% fetal bovine serum). The sample was immediately subjected to the 10X Genomics Chromium Controller with Chromium Single Cell 3′ Library & Gel Bead Kit v3.1. Libraries were constructed according to 10X Genomics Chromium Single Cell 3’ Library & Gel Bead Kit v3 protocol. Paired-end sequencing was performed by the Genomics Core Leuven on the Illumina Novaseq 6000 system. Non-fluorescent samples and samples stained with Zombie UV fixable viability stain only were prepared using the same protocol using wildtype AB zebrafish larvae for gating purposes.

### Analysis of single-cell RNA-seq data

Cell Ranger v3.0 (10X Genomics, Pleasanton, California, USA) was used for single-cell analysis to demultiplex raw base call files from Illumina sequencing and to align reads to both the zebrafish reference genome (Ensembl GRCz11.106), the HuNoV reference genome (Genbank JX459908.1), and in case of the FACS-sorted macrophages the mCherry reference sequence (X5DSL3). Cell Ranger output matrices were analyzed using the Seurat R package version 3.1 in RStudio. Cells possessing fewer than 200 unique molecular identifiers (UMIs), greater than 4,000 UMIs, or greater than 25% mitochondrial reads were removed to reduce the number of low-quality cells and doublets. Seurat was then used to normalize expression values and perform cell clustering on integrated datasets at a resolution of 0.8 with 30 principal components for the intestinal dataset and at a resolution of 0.5 with 20 principal components for the FACS-sorted macrophages. Optimal PCs were determined using JackStraw scores and elbow plots. After using the “FindMarkers” function in Seurat to identify marker genes for each cluster, clusters were annotated according to known cell type markers in zebrafish.^30^

### Gene ontology (GO) enrichment analysis

The ClueGO plug-in in Cytoscape was used for pathway analysis using a hypergeometric enrichment test.^31^ DEGs from mock- vs HuNoV-infected zebrafish leukocytes (q < 0.05), FACS-sorted macrophages (|log_2_ fold change| > 0.5 and q-value < 0.05), and all intestinal cells (|log_2_ fold change| > 0.5 and q-value < 0.05) were selected as input genes. The GO Biological Processes ontology library was used with minimum 2 genes or 2% of pathway genes (leukocytes and FACS-sorted macrophages) or minimum 3 genes or 4% pathway genes (intestinal cells) overlapping with the input DEG list, with a fusion of multiple GO parent-child terms based on similar associated genes as performed by ClueGO. Correction for multiple testing was performed by Benjamini-Hochberg. Dot plots were constructed in R using the ggplot2 function.^32^

### Chemical ablation of macrophages

Metronidazole-mediated ablation of macrophages was performed as described before.^33^ In short, zebrafish embryos of Tg(mpeg1:Gal4/UAS:nfsB-mCherry) were treated with 1 mg/mL pronase (Streptomyces griseus; Roche Diagnostics, Mannheim, Germany) to remove the chorion at 2 dpf. Next, they were placed in a solution of 5 mM metronidazole (MTZ, Sigma-Aldrich, Saint Louis, Missouri) final concentration off 1% DMSO, to induce specific ablation of NfsB:mCherry-expressing macrophages. After treatment, larvae were rinsed and placed in a 6-well plate containing fresh Danieau’s. The treatment period was either 48 h (treatment from 2 dpf until 4 dpf) or 96 h (treatment from 4 dpf until 8 dpf).

### Statistical analysis

Data was analyzed using GraphPad Prism 9 (Graph-Pad Software, San Diego, USA). Significance was determined with the nonparametric Mann-Whitney test, where ****p < 0.0001, ***p < 0.001, **p < 0.01, *p < 0.05, and *ns* is *p* ≥ 0.05. For differential expression testing of single-cell RNA-seq data, significance (*p* < 0.05) was determined in Seurat with a non-parametric Wilcoxon rank sum test with Bonferroni correction.

## Results

### HuNoV induces a strong antiviral response in gut-associated leukocytes

In response to HuNoV infection, we assessed the behavior of macrophages and neutrophils in zebrafish larvae through live imaging (Figure 1(a)), as these cells form a critical part of the innate immune system. At 2 days post-infection (dpi), both macrophages and neutrophils were recruited to the site of infection, specifically the intestines (Figure 1(b)). To quantify this response, we measured the number of macrophages and neutrophils in the whole larvae, intestines, and caudal hematopoietic tissue (CHT). Upon HuNoV infection, the number of macrophages significantly increased both in the whole larvae (Figure 1(c)) and the intestines (Figure 1(d)). Given the role of the CHT as a major hematopoietic site during zebrafish development, we investigated whether this rise in macrophages stemmed from increased hematopoiesis in this region. At 1 and 3 dpi, macrophage numbers in the CHT significantly increased (Figure 1(e)), suggesting enhanced production of macrophages at this site. For neutrophils, a significant rise was observed in the intestines at 2 dpi, which persisted through 3 dpi. Concurrently, a significant reduction in neutrophils in the CHT was noted at 2 dpi (Figure 1(h)), while overall neutrophil numbers remained stable (Figure 1(f)). This pattern indicates a redistribution of neutrophils from the CHT to the intestines, rather than an increase in production as observed for macrophages.

**Figure 2.**
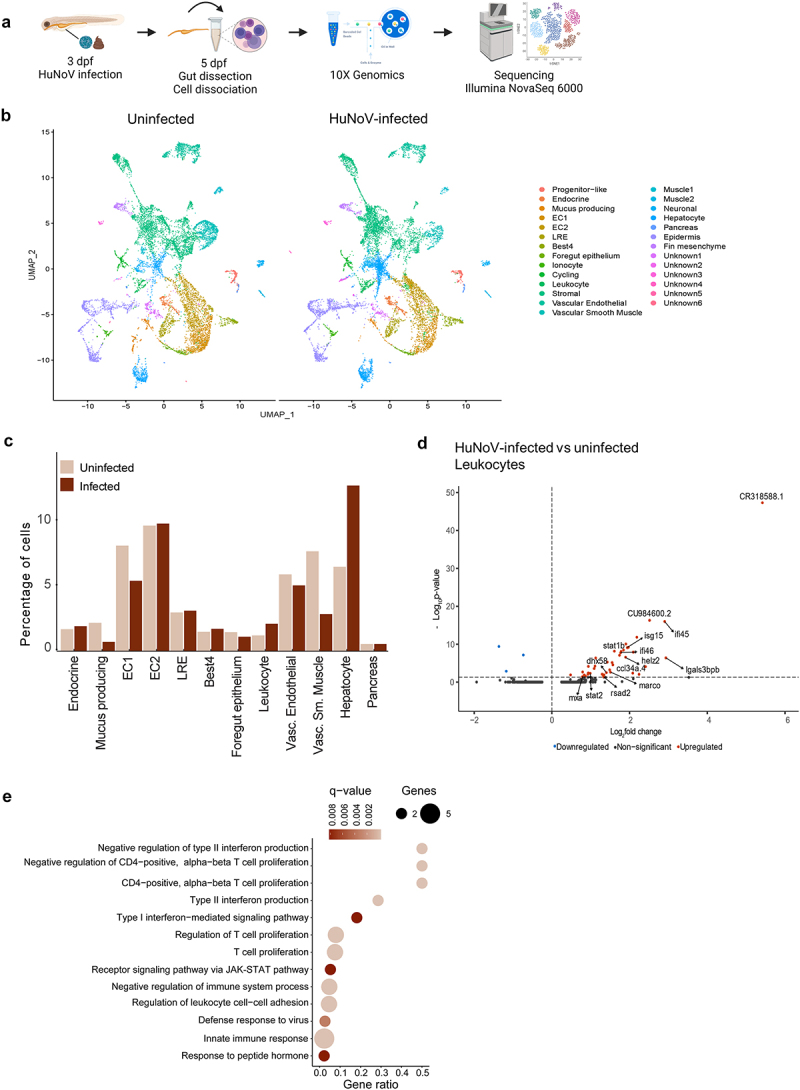
A scRNA-seq analysis on dissected zebrafish larval intestines shows an increase in leukocytes and an important role in the antiviral immune response. (a) Experimental design for transcriptional profiling of single cells in the zebrafish intestine. (b) Aggregated data of all intestinal cells (*N* = 22,958 cells (11,631 from uninfected and 11,327 from HuNoV-infected zebrafish larvae)) are represented by UMAP clustering and colored according to the unique cell cluster based on their transcriptional profile. (c) Differences in the proportions of cells between the uninfected and HuNoV-infected conditions are further displayed in detail in bar charts. (d) Volcano plots showing DEGs for the comparison of HuNoV-infected versus uninfected zebrafish larval leukocytes within the intestines. A vertical dotted line marks a log_2_(fold change) value of zero, while the horizontal dotted line marks a BenjaminiHochberg q-value of 0.05. Genes involved in relevant pathways involved in the innate immune response of antiviral signaling are displayed. (e) Dot plot showing the results of a ClueGO hypergeometric enrichment pathway analysis of upregulated DEGs using the gene ontology - biological process database. HuNoV = human norovirus, DEGs = differentially expressed genes.

To explore the cellular response of phagocytes upon HuNoV infections in depth, we analyzed single-cell transcriptomes from dissected intestines of uninfected and HuNoV-infected zebrafish larvae (Figure 2(a)). A total of 22,958 cells were clustered after filtration into 27 distinct cell types based on the expression of well-established cell type markers^30^ and included absorptive enterocytes, enteroendocrine cells, goblet cells, lysosome-rich enterocytes, Best4 cells, hepatocytes, and leukocytes (Figure 2(b), Supplementary Table S1). Examining the impact of HuNoV infection on the cellular composition revealed a reduction in mucus-producing cells (68.2%), enterocytes type 1 (EC1) (32.1%), and vascular smooth muscle cells (62.3%). Meanwhile, an increase was particularly observed for hepatocytes (96.3%) and leukocytes (75%) (Figure 2(b,c)). Within the leukocyte cell population, 42 upregulated and 3 downregulated differentially expressed genes (DEGs) were identified upon HuNoV infection (Figure 2(d), Supplementary Table S2). Leukocytes displayed an increased expression of *rsad2*, *mxa*, *dhx58*, and *helz2* genes which are linked to an antiviral immune response while also enriched in phagocytosis-linked genes including the scavenger receptors *lgals3bpb* and *marco*. Among the most highly expressed genes upon infection were the interferon-inducible genes, including *isg15* (ISG15 ubiquitin-like modifier), *ifi45* (interferon alpha-inducible protein 27.1, ifi27.1), *zgc:152791* (interferon alpha-inducible protein 27.2, ifi27.2), *CR318588.1*, and *CU984600.2*, similarly to what was recently observed in a zebrafish infection model of spring viremia of carp virus.^34^ ClueGO Gene Ontology pathway analysis on the upregulated DEGs further highlighted a role in antiviral signaling as well as in the regulation of the immune response by e.g., controlling the type II interferon production and the proliferation of CD4-positive alphabeta T cells (Figure 2(e)). Finally, the overall cellular response in the intestines was evaluated together with pathways linked to the up- and downregulated DEGs (Supplementary Figure S1, Supplementary Table S3). Altogether, we show that HuNoV induces a strong and specific antiviral immune response in zebrafish leukocytes.

### Intestinal macrophages allow HuNoV replication in zebrafish larvae

**Figure 3.**
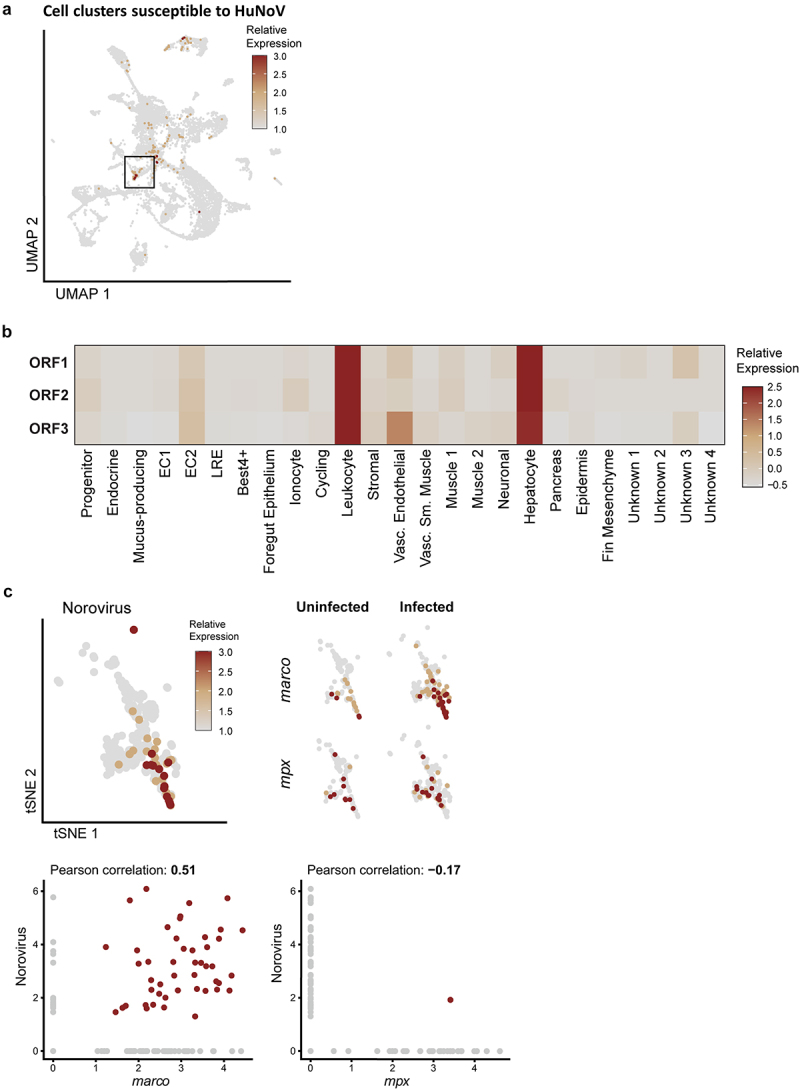
ScRNA-seq identifies enterocytes, hepatocytes, and leukocytes as host cells for HuNoV infection in the zebrafish intestines. (a) Cell clusters susceptible to HuNoV infection are highlighted and included leukocytes marked with a black box. (b) The relative expression levels of HuNoV’s ORF 1, 2, and 3 (respectively encoding for the non-structural polyprotein, VP1, and VP2) in different cell clusters are shown in detail and highlight a tropism particular for leukocytes. The average expression of each norovirus read per cell type was determined followed by a normalization across all cell types. (c) Within leukocytes, a 2D t-SNE projection of the leukocyte cell cluster shows the relative expression of HuNoV. Within leukocytes, the expression of *marco*, i.e., a marker for macrophages *and mpx*, i.e., a marker for neutrophils is shown in both uninfected and HuNoV-infected conditions. The expression of HuNoV and the macrophage marker *marco* show an overlap corresponding to a Pearson correlation coefficient of 0.51. In contrast, no overlap is seen between the expression of *mpx*, a marker for neutrophils corresponding to a low Pearson correlation coefficient of − 0.17. HuNoV = human norovirus, ORF = open reading frame, VP1 = viral protein 1, major capsid protein, VP2 = viral protein 2, minor capsid protein, dsRNA = double-stranded RNA.

To investigate the *in vivo* host cell tropism of HuNoV in the intestine in an unbiased manner, norovirus transcripts of open reading frame (ORF) 1, 2, and 3 (respectively encoding for the non-structural polyprotein, VP1, and VP2) were mapped to scRNA-seq-derived intestinal cell clusters (Figure 3(a,b)). A detection of high levels of all 3 ORFs is suggestive for a productive infection^35^ and was observed for enterocytes type 2 (EC2), leukocytes, and hepatocytes. Among leukocytes, macrophages showed high norovirus expression (Pearson coefficient = 0.51), while neutrophils exhibited no expression overlap (Pearson coefficient = −0.17) (Figure 3(c)). To date, functional lymphocytes and dendritic cells have only been described in adult zebrafish^36,37^ wherefore expression of HuNoV in these cell types was not further investigated.

**Figure 4.**
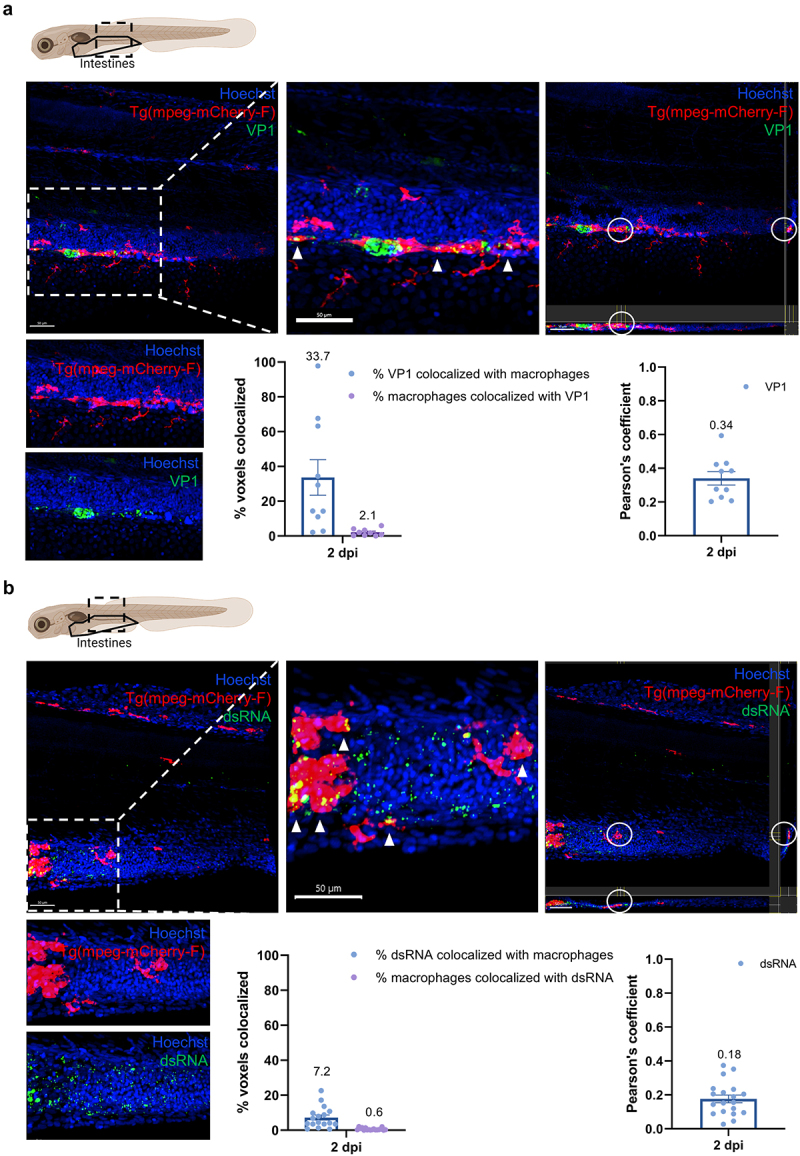
Macrophages in the intestines are infected by HuNoV and allow active viral replication. (a-b) whole mount immunohistochemistry confocal images of Tg(mpeg:mCherry-F) zebrafish larvae infected with HuNoV taken at 2 dpi at a 25X magnification focusing on the intestines using Hoechst, a primary mCherry antibody, and (a) a VP1-targeting antibody or (b) a dsRNA-targeting antibody. Cross-section views created in imaris software show horizontal and vertical sections of macrophages that contain (a) VP1 or (b) dsRNA with the white circle highlighting an infected cell of interest. Imaris 3D Colocalization software calculated the overlap of voxels between macrophages and (a) VP1 or (b) dsRNA (*N* = 10-19) together with a thresholded Pearson coefficient, mean values ± SEM are shown. HuNoV = human norovirus, dpi = days post infection, VP1 = viral protein 1, major capsid protein, dsRNA = double-stranded RNA, SEM = standard error of means.

Confocal imaging of HuNoV-infected Tg (mpeg:mCherry-F) zebrafish larvae further confirmed an infection of intestinal macrophages at the peak of viral replication, i.e., 2 dpi (Figure 4(a), Supplementary Figure S2a, and S3a). Using Imaris 3D colocalization software, we determined that 33.7% of the thresholded capsid protein VP1 signal colocalized with intestinal macrophages, while 2.1% of the thresholded intestinal macrophage signal overlapped with VP1 signal (Pearson coefficient of 0.34).

We next evaluated whether HuNoV was present within macrophages due to phagocytosis, or whether HuNoV can actively replicate in intestinal macrophages. Similar to VP1, double-stranded RNA (dsRNA), the RNA intermediate that is produced during active viral replication, was present in a distributional pattern across the intestines and was detected within intestinal macrophages (Pearson coefficient of 0.18) (Figure 4(b), Supplementary Figure S2b, and S3b). In addition, triple staining’s of macrophages, VP1, and dsRNA were performed, which were all found to colocalize (Supplementary Figure S4). This indicates that HuNoV actively replicates in intestinal macrophages.

In addition to the intestines, we previously identified HuNoV capsid protein in the CHT region of zebrafish larvae.^5^ At 2 dpi, HuNoV VP1 was identified within macrophages in the CHT (Supplementary Figure S5a, S2c, and S3c). Colocalization between VP1 and macrophages was 58.1%, i.e., more than half of the thresholded VP1 signal in the CHT was within macrophages; whereas 11.7% of the thresholded macrophage signal colocalized with VP1 signal (Pearson coefficient 0.46). In contrast to viral structural protein, confocal imaging identified no dsRNA intermediate signal in the CHT at 2 dpi, and thereby not within macrophages in this tissue (Supplementary Figure S5b and S2d). Likewise, at 1 and 3 dpi no dsRNA signal was detected within the CHT, excluding this was due to variable viral replication kinetics (Supplementary Figure S6). This leads us to conclude that it is unlikely that there is HuNoV replication in macrophages residing in this tissue.

**Figure 5.**
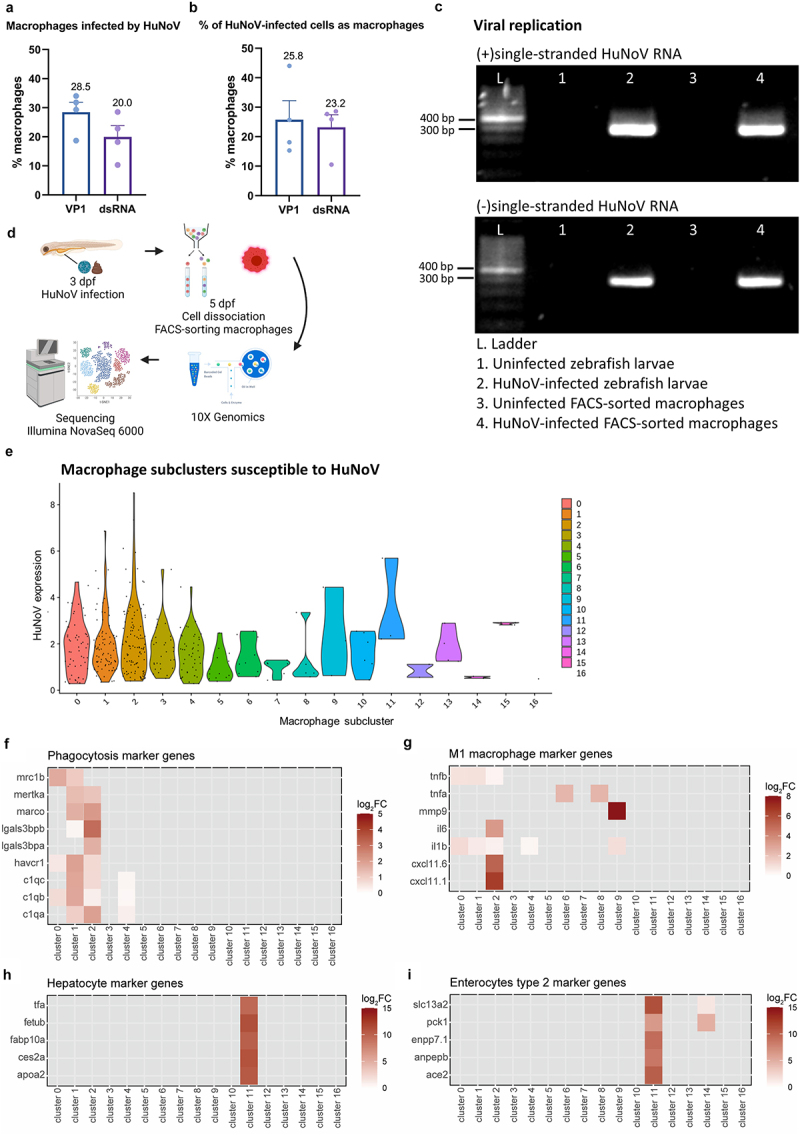
The *in vivo* tropism for macrophages is further confirmed by combined results of flow cytometry, RT-PCR on FACS-sorted macrophages, and a scRNA-seq analysis on dissected zebrafish larval intestines. Flow cytometry analysis on 2 dpi Tg(mpeg:mCherry-F) zebrafish larvae infected with HuNoV shows (a) the percentage of macrophages that contain HuNoV (either VP1 or dsRNA) and (b) the percentage of overall infected cells (VP1 or dsRNA) that are identified as macrophages. *N* = 4. Mean values ± SEM are shown. (c) Detection of (+)ss and (-)ss HuNoV RNA in both whole zebrafish larvae and FACS-sorted macrophages from HuNoV-infected Tg(mpeg:mCherry-F) zebrafish larvae. Strand-specific RT-PCR shows that (-)ss HuNoV RNA was detected intracellularly in macrophages at 2 dpi. (d) Experimental design for transcriptional profiling of FACS-sorted macrophages. (e) Violin plots show the normalized HuNoV expression per cell per macrophage subcluster. Heatmaps show the expression of genes linked to (f) phagocytosis, (g) M1 inflammatory macrophages, (h) hepatocytes, and (i) enterocytes type 2 across the macrophage subclusters. HuNoV = human norovirus, VP1 = viral protein 1, major capsid protein, dsRNA = double-stranded RNA, ss = single-stranded.

Next, we evaluated the percentage of infected macrophages (Figure 5(a)), as well as the relative overall importance of macrophages as host cells (Figure 5(b)) by flow cytometry. At the whole organism level, 28.5 ± 3.4% of the macrophages contained viral capsid protein, while 20.0 ± 3.8% contained the dsRNA intermediate (Figure 5(a)). Of all HuNoV-positive cells at the peak of infection, 25.8 ± 6.5% of VP1-positive cells and 23.2 ± 4.3% of dsRNA-positive cells were macrophages (Figure 5(b)). Flow cytometry gating strategies and specificity of the VP1 and dsRNA antibodies are shown in Supplementary Figure S7-S8.

As HuNoV, a (+)ssRNA (single-stranded) virus, replicates through a (-)ssRNA intermediate, the presence of (-)ssRNA within macrophages is an additional layer of proof of active viral replication within this cell type. A strand-specific RT-PCR on a single-cell suspension of FACS-sorted macrophages from infected Tg (mpeg:mCherry-F) larvae was performed at 2 dpi. Both (+)ss and (-)ss HuNoV RNA were detected in FACS-sorted macrophages (as well as in whole larvae), thereby confirming active viral replication in macrophages (Figure 5(c)).

Next, we examined the single-cell transcriptomes of macrophages isolated from HuNoV-infected Tg (mpeg:mCherry-F) larvae using FACS (Figure 5(d) and Supplementary Figure S9). Sixteen distinct macrophage clusters were identified (Supplementary Table S4). HuNoV was detected within specific macrophage subclusters, with the highest expression of HuNoV reads observed in clusters 0–4 (Figure 5(e)). In these clusters, the expression of marker genes linked to phagocytosis and pro-inflammatory M1 macrophage markers^38^ was higher compared to other clusters with fewer or no viral reads detected (Figure 5(f,g)). Previous studies have proposed that HuNoV detection in macrophages is due to phagocytosis of infected intestinal epithelial cells.^8,9^ Our findings challenge this hypothesis. Markers for enterocytes type 2 and hepatocytes, identified here as the primary host cells for HuNoV alongside macrophages, are highly expressed in clusters 11 and 14 containing only a negligible portion of norovirus reads (Figure 5(e,h-i)). This indicates that the presence of HuNoV in macrophages is not solely a result of phagocytosis of infected intestinal epithelial cells.

### Single-cell analysis reveals inhibition of cytoplasmic translation and enhanced phagocytosis and apoptosis in HuNoV-infected macrophages

**Figure 6.**
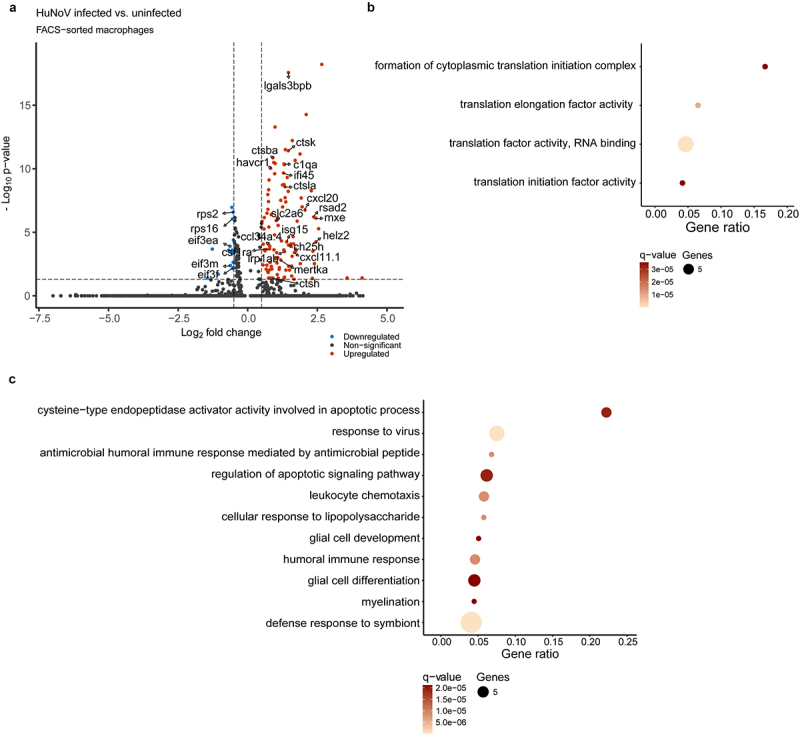
Single-cell analysis reveals inhibition of cytoplasmic translation and enhanced phagocytosis in HuNoV-infected macrophages. (a) A volcano plot shows the DEGs in HuNoV-infected vs. uninfected bystander macrophages within the HuNoV-infected zebrafish host. A vertical dotted line marks a log_2_(fold change) value of 0.5, while the horizontal dotted line marks a benjamini-Hochberg q-value of 0.05. Dot plot showing the pathways obtained via a ClueGO hypergeometric enrichment pathway analysis of (b) downregulated and (c) upregulated DEGs using the gene ontology - biological process database. HuNoV = human norovirus, DEGs = differentially expressed genes.

Furthermore, single-cell resolution analysis enhances our understanding of the differential responses between directly infected and bystander-activated macrophages within the host. Upon infection, we identified 17 downregulated and 112 upregulated differentially expressed genes (Figure 6(a), Supplementary Table S5). Notably, many downregulated genes, including *rsp2*, *rsp16*, *eif3f*, *eif3ea*, and *eif3m*, are involved in cytoplasmic translation (Figure 6(a,b)), suggesting significant inhibition of this process in HuNoV-infected macrophages compared to bystander macrophages. Viruses, as intracellular pathogens, rely on the host’s translational machinery for protein production, and inhibition of this process is a common antiviral mechanism used by interferon-stimulated genes. A broad spectrum of these interferon-stimulated genes, such as *isg15*, *rsad2*, *helz2*, *mxe*, and *ifi45*, are upregulated in HuNoV-infected macrophages, contributing to the innate defense response (Figure 6(a,c)). Additionally, the upregulated expression of genes including *ch25h*, *ccl34a.4*, *csf1ra*, and *cxcl20* is associated with leukocyte chemotaxis and the humoral immune response (Figure 6(a,c)). Upon infection, macrophages show a significant upregulation of phagocytosis-associated genes, including scavenger receptors (*lgals3bpb* and *marco*), the phosphatidylserine receptor *havcr1*, complement component *c1qa*, and pro-phagocytic receptors *lrp1ab* and *mertka* (Figure 6(a)). Additionally, an upregulation of the cysteine cathepsin genes *ctsh*, *ctsba*, *ctsk*, and *ctsla* was noted (Figure 6(a)). Cathepsins play an important role in antigen presentation in macrophages via phagosomes^39^ and act as upstream activators of the intrinsic apoptotic pathway (Figure 6(c)). Finally, an upregulation of the solute carrier family 2 member 6 *slc2a6* (GLUT6) encoding for a glucose transporter was noted upon HuNoV infection (Figure 6(a)).

### The role of neutrophils in HuNoV infection is limited

ScRNA-seq analysis of dissected intestines of HuNoV-infected zebrafish hinted a limited role for neutrophils in HuNoV infections. Confocal microscopy of Tg(mpx:GFP) zebrafish larvae stained for VP1 confirmed this by a limited colocalization (Pearson coefficient of 0.15) of both within the intestine (Supplementary Figure S10a, S11a, and S11e). Additionally, limited to no colocalization was found between neutrophils and dsRNA (Pearson coefficient close to zero) (Supplementary Figure S10b, S11b, and S11d). In the CHT, neutrophils also did not colocalize with HuNoV capsid protein (Supplementary Figure 10C and 11C) nor with dsRNA (Supplementary Figure S10d and S11d).

### Macrophages are not essential for HuNoV replication or clearance in the zebrafish larval model

**Figure 7.**
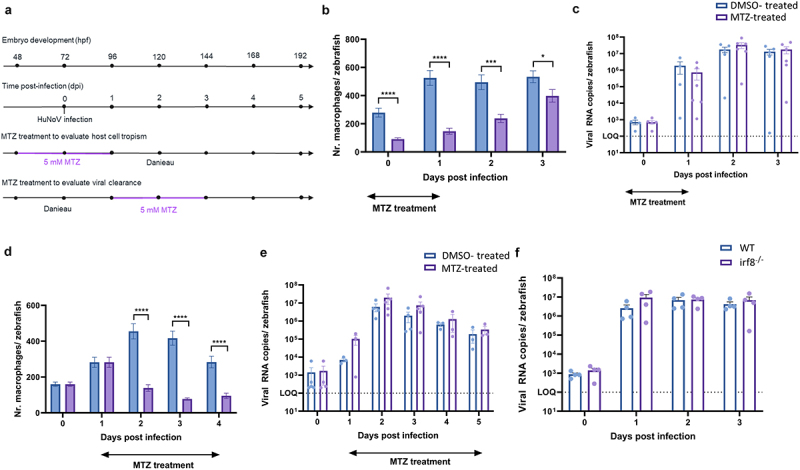
Macrophages are not crucial for either viral replication or viral clearance. (a) Graphical presentation of MTZ treatment schedule before and after infection. (b, d) bars represent the number of macrophages per zebrafish larva of Tg(mpeg1:Gal4/UAS:nfsB-mCherry) quantified using ImageJ software when larvae are treated with DMSO or MTZ to induce chemical ablation of macrophages. *N* = 18-29 zebrafish larvae per group. (c, e) bars represent HuNoV viral RNA copies per zebrafish larva quantified using RT-qPCR. *N* = 3-5 independent experiments with 10 zebrafish larvae per group. (b-e) mean values ± SEM are shown. The dotted line shows the LOQ. (f) Bars represent HuNoV viral RNA copies per WT or irf8^−/−^ zebrafish larva using RT-qPCR. *N* = 4 independent experiments with 10 zebrafish larvae per group, mean values ± SEM are shown. The dotted line shows the LOQ. For all graphs: outliers were removed (ROUT, Q = 1%). Statistical analysis was performed using the Mann-Whitney test. Significantly different values are indicated by asterisks; *p < 0.05, ***p < 0.001, ****p < 0.0001. (b) Before HuNoV infection, MTZ treatment reduced macrophage numbers by 67% (*p* < 0.0001) at 0 dpi, by 72% (*p* < 0.0001) at 1 dpi, by 52% (*p* = 0.0003) at 2 dpi, and by 25% (*p* = 0.0449) at 3 dpi. (d) After HuNoV infection, MTZ treatment reduced macrophage numbers by 70% (*p* < 0.0001) at 2 dpi, by 82% (*p* < 0.0001) at 3 dpi, and by 67% (*p* < 0.0001) at 4 dpi. MTZ = metronidazole, DMSO = dimethylsulfoxide, HuNoV = human norovirus, hpf = hours post fertilization, dpi = days post infection, WT = wildtype, KO = knockout, SEM = standard error of means, LOQ = limit of quantification.

To evaluate whether macrophages are necessary for a productive HuNoV replication, we used the transgenic zebrafish line Tg (mpeg1:Gal4FF/UAS:NfsB:mCherry), in which macrophages can be selectively ablated by metronidazole (MTZ) treatment (Figure 7(a)). MTZ treatment before HuNoV infection (i.e., at 48 hpf) significantly reduced the number of macrophages each dpi (Figure 7(b) and Supplementary Figure S12a-b). This reduction in the number of macrophages, and thus host cells, led to no significant reduction in HuNoV viral load (Figure 7(c)). MTZ treatment after HuNoV infection (i.e., at 96 hpf/1 dpi) significantly reduced macrophage numbers from 2 dpi onwards (Figure 7(d) and Supplementary Figure S12c-d). There was however no significant impact on HuNoV viral load or on the duration of the infection, hinting that macrophages are not the only cell type involved in (or capable of) clearing a HuNoV infection in zebrafish larvae (Figure 7(e)). MTZ treatment did not affect HuNoV replication itself, as shown by replication in wildtype MTZ-treated zebrafish larvae (Supplementary Figure S12e-f). Since MTZ treatment reduces macrophage numbers only transiently, we additionally infected irf8^−/−^ larvae, a transgenic line that completely lacks macrophages in the larval stage. Compared to wildtype zebrafish larvae of the same developmental stage, no significant difference was observed in HuNoV replication confirming the hypothesis that macrophages are not necessary for viral replication or infection control (Figure 7(f)).

## Discussion

HuNoVs have historically posed challenges for cultivation in cell culture, but recent breakthroughs, including infection of differentiated HIEs and a subset of established human B cell lines,^4,10,11^ have provided insights into the *in vitro* cellular tropism and the potential overlap with that of MNV. The question of whether the *in vitro* cellular tropism of HuNoV indeed includes both intestinal epithelial cells and immune cells, and whether this is found also in an *in vivo* setting remained to be thoroughly addressed. Previously, large animal models, including chimpanzees and gnotobiotic pigs, suggested the infection of macrophages, dendritic cells, and lymphocytes.^12,13^ However, these larger animal models are less amenable to extensive studies and are less in compliance with current ethical regulations. Rag^−/−^ γc^−/−^ BALB/c mice could support HuNoV replication, albeit with limited application potential due to a short-lived replication that was cleared within 72 hours.^40^ In the pursuit of a small animal model supporting a more efficient HuNoV replication, we have developed a zebrafish larval model. Zebrafish, while genetically more distinct from humans in comparison to chimpanzees, pigs, and mice, have recently gained prominence in biomedical research.^41^ The use of zebrafish larvae offers several advantages, including their small size, optical transparency, and ease of generating transgenic reporter lines.^21^ In the current study, we used these strengths to address existing knowledge gaps pertaining to HuNoV cell tropism and host-pathogen interactions.

Neutrophils and macrophages are the early responders to infection in zebrafish larvae, alike in humans.^42,43^ Using live imaging, we demonstrated an overall increase in macrophage numbers, along with the recruitment of both macrophages and neutrophils to the intestines following HuNoV infection. The innate immune response, encompassing the production of interferons and downstream interferon-stimulated genes, plays a pivotal role in the initial defense against invading pathogens which is highly conserved between fish and humans.^44^ Gene Ontology analysis revealed an immune response within the intestines of HuNoV-infected zebrafish larvae, primarily characterized by type I interferon-mediated signaling via the JAK-STAT pathway, consistent with findings in HuNoV-infected jejunal and terminal ileum HIEs.^45,46^ Besides our understanding of the innate immune response by intestinal epithelial cells, we here have an opportunity to study the role of many other cell types, including the key innate immune cells, in more detail. Leukocytes, with macrophages and neutrophils as the major players of the innate immune system, are the primary phagocytes responsible for pathogen destruction after uptake. Utilizing scRNA-seq, we observed an antiviral response via the JAK-STAT pathway to manage the infection, while also contributing to immune response control, such as regulating type II interferon production and CD4-positive, alpha-beta T cell proliferation.

Previously, we identified the intestines as primary sites of HuNoV infection^5^ and noted the recruitment of both macrophages and neutrophils to this tissue. Next, we aimed to determine whether some of the infiltrating immune cells not only participate in the immune response and viral clearance but also become directly infected by HuNoV. Using scRNA-seq of dissected intestinal tissues, we unbiasedly deciphered the cellular tropism of HuNoV. Our results revealed high levels of all three ORFs within individual enterocytes, hepatocytes, and notably, macrophages, indicating productive infection in these cell types.

Interestingly, studies in immunocompromised patients^8,9^ and various animal models^13,40,47^ have provided evidence for the presence of HuNoV proteins within macrophages in the lamina propria. However, it remained unclear whether these proteins result from phagocytosis of infected epithelial cells or from active viral replication. Through immunofluorescence staining, flow cytometry, and negative sense RT-PCR on FACS-sorted macrophages, we conclusively identified macrophages (in contrast to neutrophils) as permissive immune cells for both HuNoV infection and replication. Using an additional round of scRNA-seq on FACS-sorted macrophages from HuNoV-infected zebrafish, we determined the host gene correlates of HuNoV infection. HuNoV was found to preferentially infect macrophage cell clusters expressing phagocytosis and inflammatory M1 marker genes. The mechanism of HuNoV entry into host cells, including whether this process is receptor-based, phagocytosis-based, or a combination of both, remains unknown. However, selective infection of highly phagocytosing macrophages suggests that phagocytosis could be involved in cell entry.

Peiper et al.^48^ previously challenged the belief that HuNoV replication within macrophages is solely the result of replicating virus originating from phagocytosed intestinal epithelial cells. It was pointed out that similar levels of intestinal epithelial cell markers were measured in macrophages from either mock- or HuNoV-infected patient biopsies.^8^ In this study, we provide the missing evidence as we identified macrophage subclusters expressing marker genes for HuNoV host cells, i.e., enterocytes and hepatocytes, but these subclusters contained a negligible fraction of norovirus reads. Consequently, the identification of all three ORFs of HuNoV combined with the detection of both dsRNA and (-)ssRNA in macrophages can be attributed to a direct infection. Neutrophils, which are known to phagocytose infectious cellular debris,^49^ showed no correlation with HuNoV viral reads via scRNA-seq combined with the lack of colocalization observed through whole mount immunofluorescence. This dataset supports the conclusion that, in the zebrafish model, macrophages serve as host cells for HuNoV, facilitating an active replication cycle. Since zebrafish are not the natural host for HuNoV infections, the identified cellular tropism should still be confirmed in the human host.

The detection of conserved viral nucleic acids, including dsRNA, within macrophages triggers a robust interferon-mediated immune response, characterized by a significant upregulation of interferon-inducible genes. HuNoV-infected macrophages exhibited increased expression of the prophagocytic receptor *lrp1ab* and the phosphatidylserine receptor *havcr1* compared to bystander-activated macrophages. LRP1 was recently identified as a proteinous host factor acting as a receptor for Rift Valley fever virus^50^ and Oropouche orthobunyavirus,^51^ whereas SARS-CoV-2 engaged LRP1 in the late stages of intracellular replication.^52^ Moreover, the hepatitis A virus cellular receptor 1

(HAVCR1/TIM-1) serves as a receptor of various viruses, facilitating viral infection.^53–55^ The potential mechanisms of norovirus entry into macrophages, whether through phagocytosis or via a proteinous receptor, warrant further investigation. Furthermore, the professional phagocytes exhibit increased expression of cysteine cathepsin H, B, K, and L, enzymes involved in apoptosis. Cathepsin B-mediated intrinsic apoptosis has been observed *in vitro* with MNV,^56^ and this virus-induced programmed cell death has been linked to the release of infectious viruses from macrophages.^57^ Additionally, HuNoV-infected macrophages show elevated expression of *merkta* and the complement component *c1qa*, both of which facilitate phagocytosis of apoptotic cells by macrophages.^58,59^ Investigating whether HuNoV, akin to other non-enveloped viruses, employs regulated cell disassembly for vesicle-associated egress could reveal mechanisms of immune evasion and phagocytic uptake by new target cells.^60^ Moreover, cathepsin L is implicated in the entry and uncoating of virus capsid proteins for several caliciviruses,^61^ suggesting a potential role in the infection dynamics of HuNoV. Finally, HuNoV infection in macrophages leads to increased expression of the glucose transporter GLUT6, encoded by the *slc2a6* gene. A similar upregulation of the lysosomal glucose transporter SLC2A6 was observed in mouse macrophages upon inflammatory stimuli, which results in an energy metabolism shift favoring glycolysis.^62^ Glycolysis has been identified as a crucial factor for MNV replication in macrophages *in vitro* ,^63^ but its role in HuNoV infection remains to be elucidated. Subsequent studies using zebrafish knockout models should focus on the importance of the identified pathways in HuNoV replication and pathogenesis. A better understanding of the antiviral response could open up new avenues for the development of efficient host-targeting therapeutics and aid vaccine development.

The results of this study were insufficient to determine whether infectious viral progeny was released from macrophages following HuNoV replication. It would be very challenging to culture FACS-sorted zebrafish larval macrophages for sufficient days to perform infection studies on HuNoV replication, particularly given such cultures are kept at 28°C.^64^ Given the differentiated state of macrophages, they are unable to proliferate and be subcultivated; no monocyte zebrafish cell line is currently available to our knowledge.

Importantly, all infections were carried out using a HuNoV GII.4 Sydney [P4], the predominant genotype worldwide. However, HuNoVs exhibit great genetic diversity.^65^ Variations in capsid proteins among different genotypes could potentially influence host cell interactions and eventually virus entry, affecting cellular tropism across HuNoV genotypes. Furthermore, it should be investigated whether the higher virulence of GII.4 strains can be attributed to differences in cell tropism. Infection and replication within macrophages, which circulate in the bloodstream, may represent a rapid route for viral dissemination, potentially contributing to the heightened virulence of macrophage-tropic strains. Whether macrophage infection facilitates HuNoV extraintestinal dissemination and persistence within the host is another aspect worthy of investigation as chronic HuNoV infections are highly debilitating for immunocompromised patients.

Macrophages are key immune players but also provide additional support in the tissue they are residing in.^66^ An interesting research direction involves the bidirectional communication between the enteric nervous system (ENS) and local immune players. Further research is needed to study the activation of macrophages by HuNoV infection and the influence hereof on the intestinal peristaltic movements regulated by the ENS, which relates to HuNoV pathogenesis.

In this research, it was determined through a transient chemical ablation and an irf8^−/−^ zebrafish model that macrophages are dispensable for sustaining a productive HuNoV infection. ScRNA-seq analysis on the zebrafish intestines pointed toward the ability of the virus to infect different cellular types to achieve a productive infection and thus, in the absence of macrophages, other cells could further support replication. Whether they play an indispensable role in viral dissemination beyond the intestinal epithelium or in viral pathogenesis should be addressed in future research.

Interestingly the irf8^−/−^ zebrafish larvae display a compensatory increase in neutrophils.^67^ This raises the question of whether neutrophils become more crucial in the control (i.e., by taking over the role of macrophages in their absence) of HuNoV and perhaps other viral infections,^16,68^ a topic worthy of further investigation.

Moving forward, exploring the role of additional host cells identified here by scRNA-seq presents a promising avenue. Interestingly, we identified all three ORFs of HuNoV within hepatocytes through scRNA-seq analysis. This observation aligns with previous findings of HuNoV presence in liver tissue in various models, including zebrafish, chimpanzees, and Rag^−/−^γc^−/−^ BALB/c mice.^5,12,40^ Hepatocytes are responsible for the production of bile acids, which were previously shown to be essential for the replication of HuNoV GII.3, while supporting the replication of HuNoV GII.4 in HIEs.^69^ Since the majority of bile acids is returned to the liver via the enterohepatic circulation,^70^ it should be investigated how HuNoV disseminates to extraintestinal tissues including the liver. Given the promising similarities between the zebrafish and human liver in terms of structure, function, and regenerative capacity, the zebrafish model offers an intriguing platform for further investigating the precise role of hepatocytes in acute HuNoV infections.^71^

ScRNA-seq revealed a significant reduction in the number of both enterocyte type 1 and goblet cells. The mechanism by which HuNoV exits host cells, whether through lytic infection^72^ or a vesicle-mediated non-lytic process,^73^ remains uncertain. The observed decrease in cell numbers may be due to lytic infection, potentially leading to an underestimation of their role as HuNoV host cells. This warrants further investigation in future studies.

Overall, we showed that macrophages are infected by HuNoV while supporting active viral replication in the zebrafish larval model. This supports the idea that HuNoV and MNV share, at least to a certain degree, a dual cell tropism for both intestinal epithelial and myeloid cells. Understanding the *in vivo* cellular tropism of HuNoV will advance the development of more efficient and robust cultivation systems, facilitating the generation of cell culture-derived virus stocks. Such advancements would be invaluable for both basic and translational research, paving the way for the development of effective antiviral treatments and vaccines, which are currently lacking.

## Supplementary Material

Supplemental Material

## Data Availability

The single-cell RNA-seq data discussed in this publication have been deposited in NCBI’s Gene Expression Omnibus (Edgar et al., 2002) and are accessible through GEOSeriesaccessionnumberGSE261163 (https://www.ncbi.nlm.nih.gov/geo/query/acc.cgi?acc=GSE261163). Any additional information required to reanalyze the data reported in this paper is available upon request to the corresponding author.
