# Supplementary figures and images for: Transcriptional profiling of zebrafish intestines identifies macrophages as host cells for human norovirus infection
Source: Gut Microbes. 2024 Nov 25;16(1):2431167. doi: 10.1080/19490976.2024.2431167 (PMC11591593; doi:10.1080/19490976.2024.2431167)

Fig s7


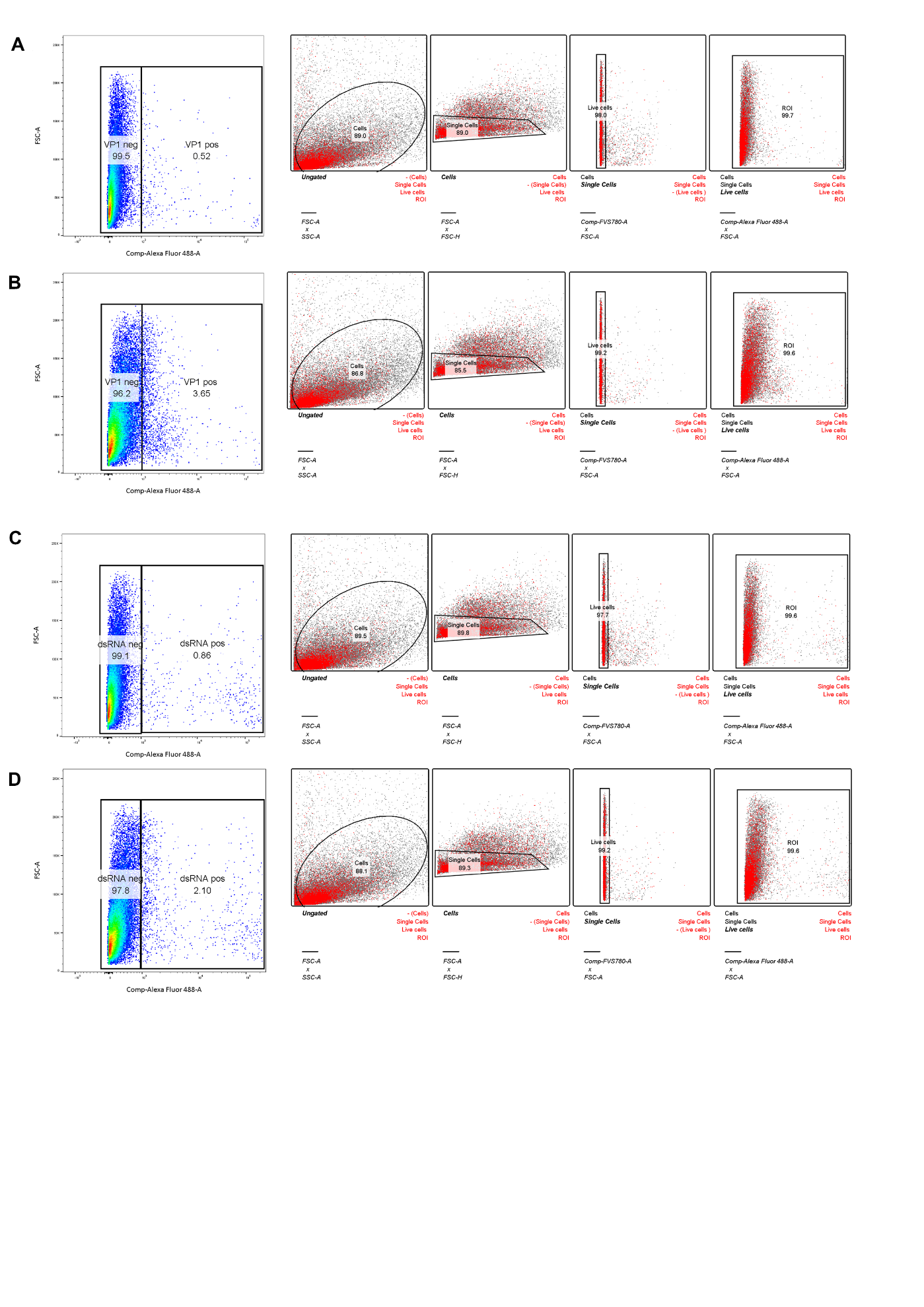


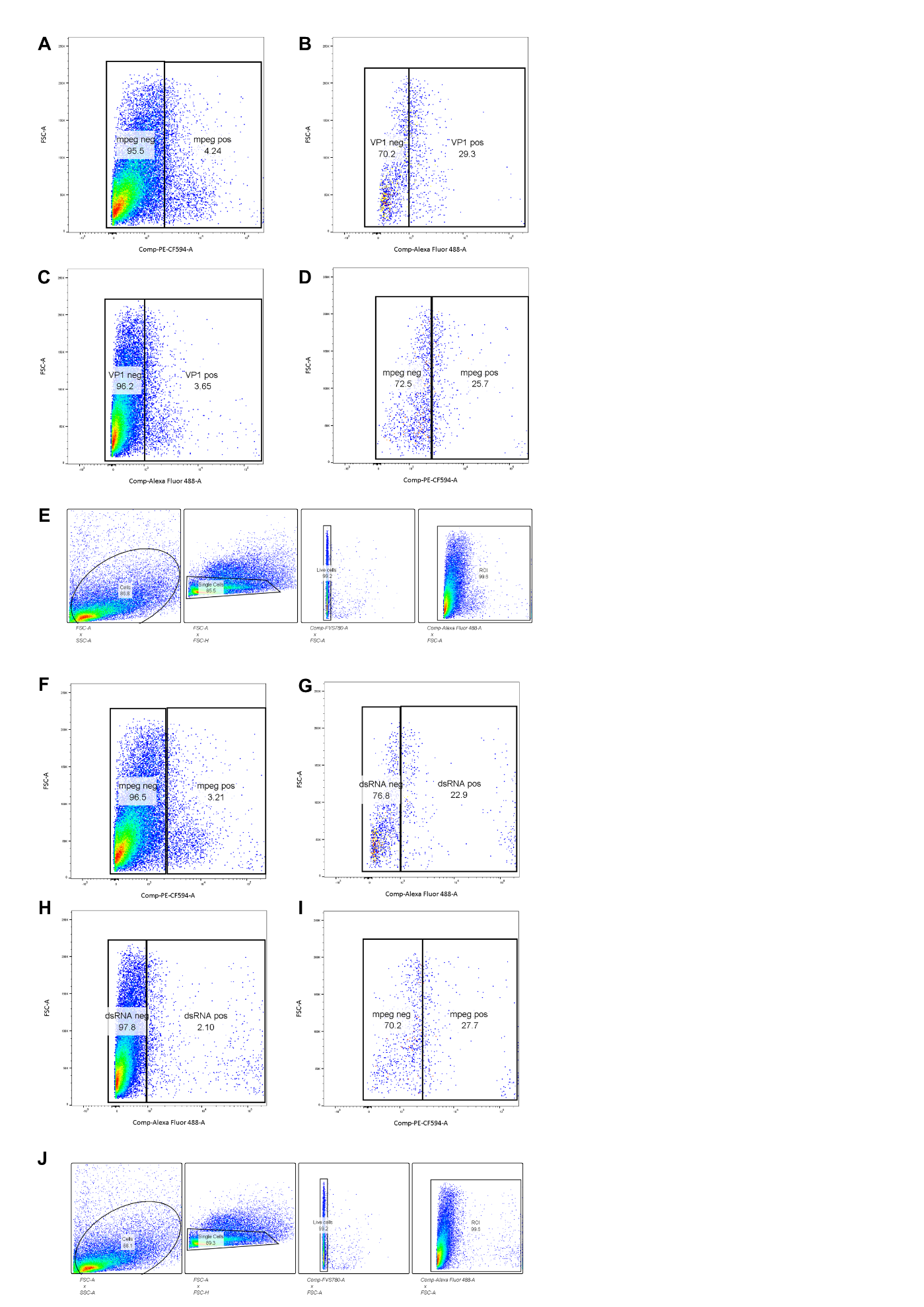
Fig s8

Fig s9


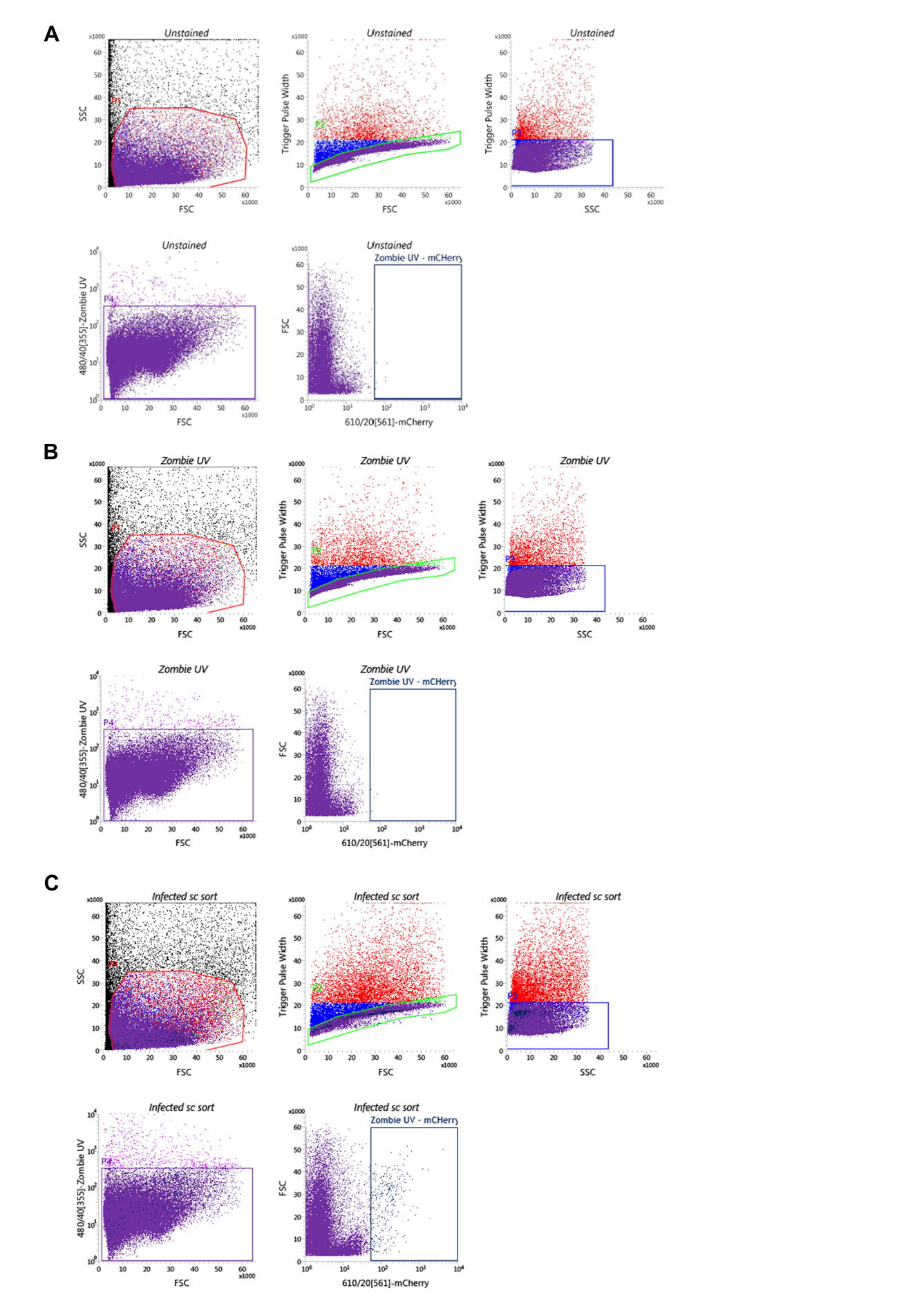


Fig s10


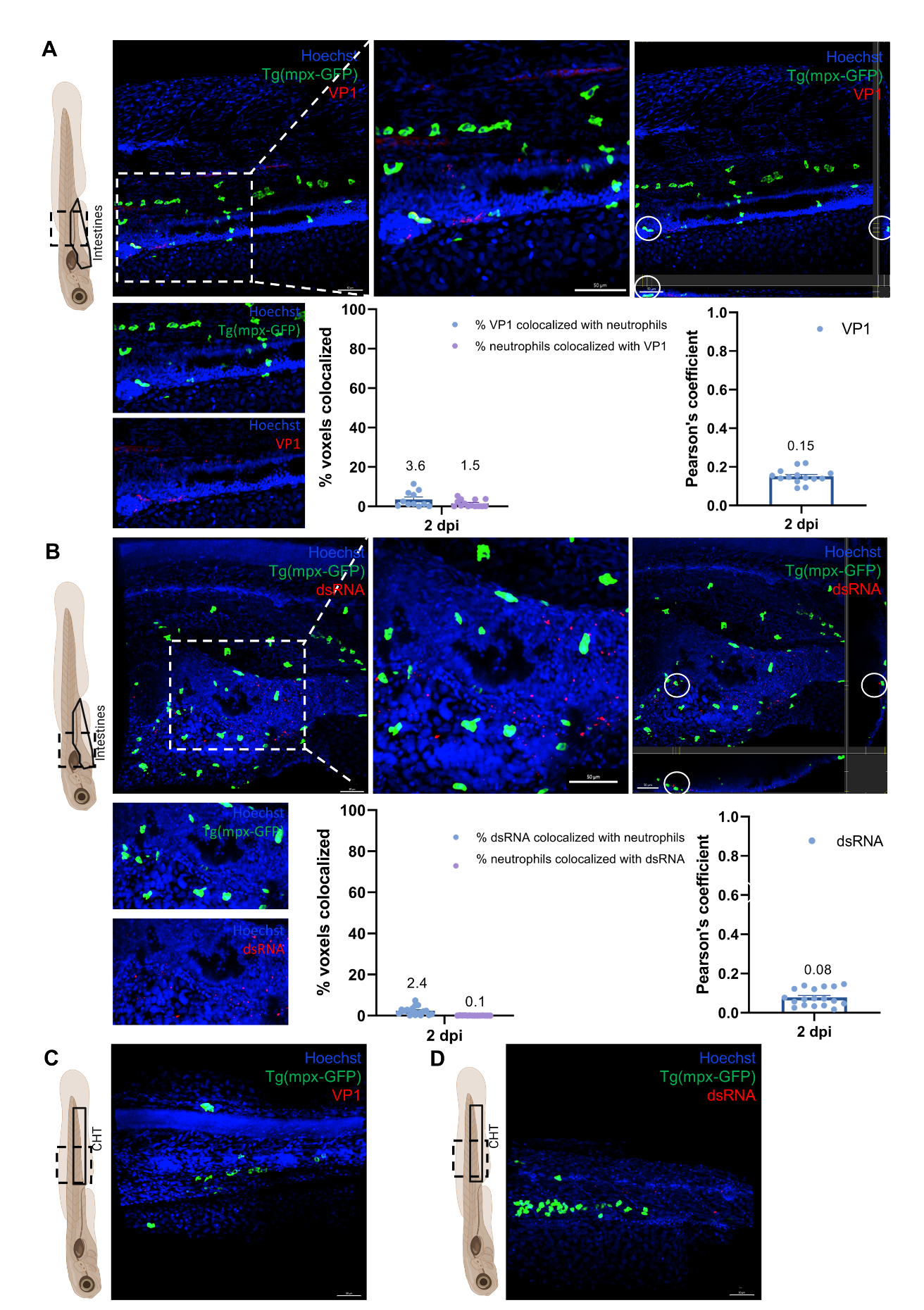


Fig s11


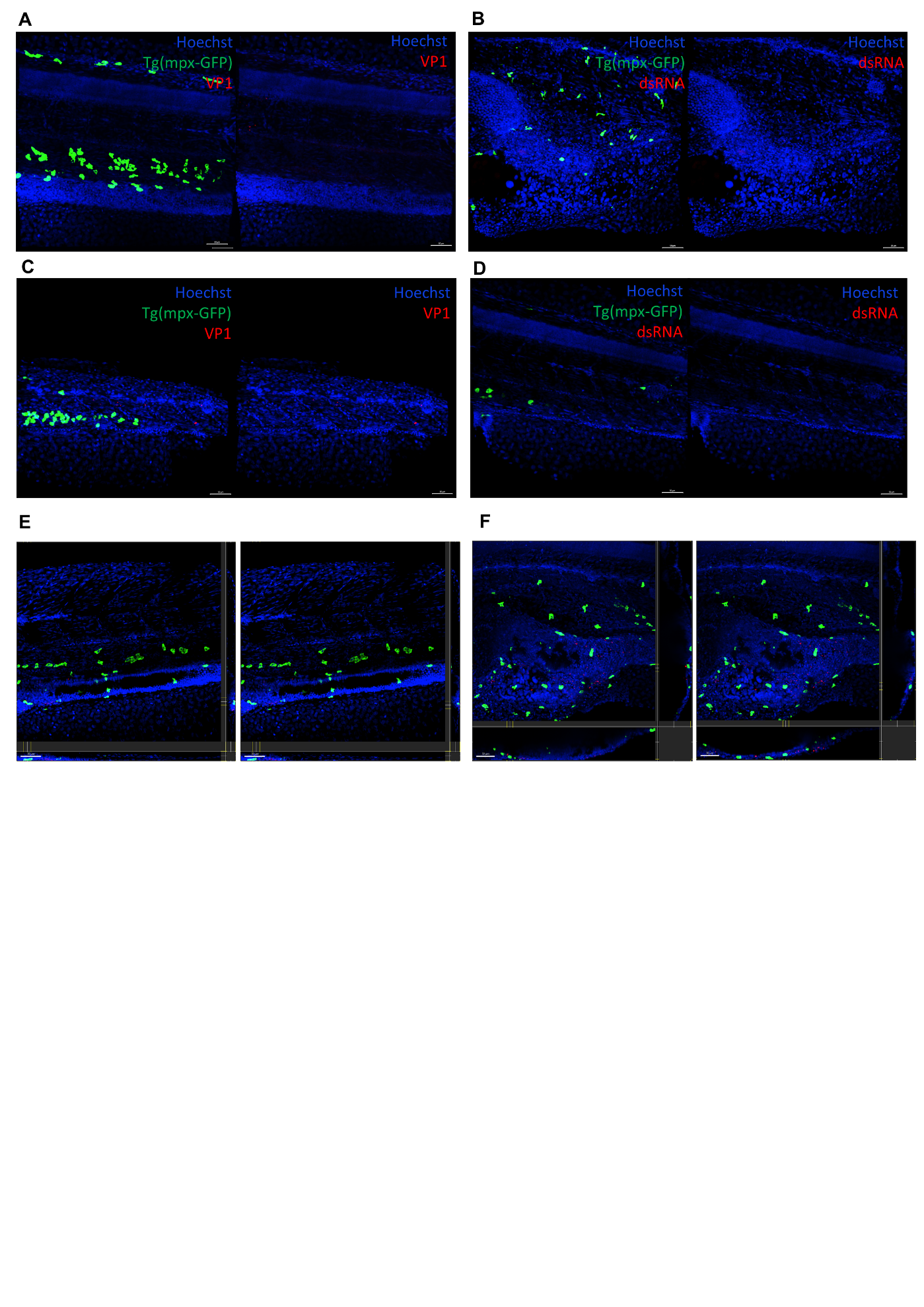


Fig s12


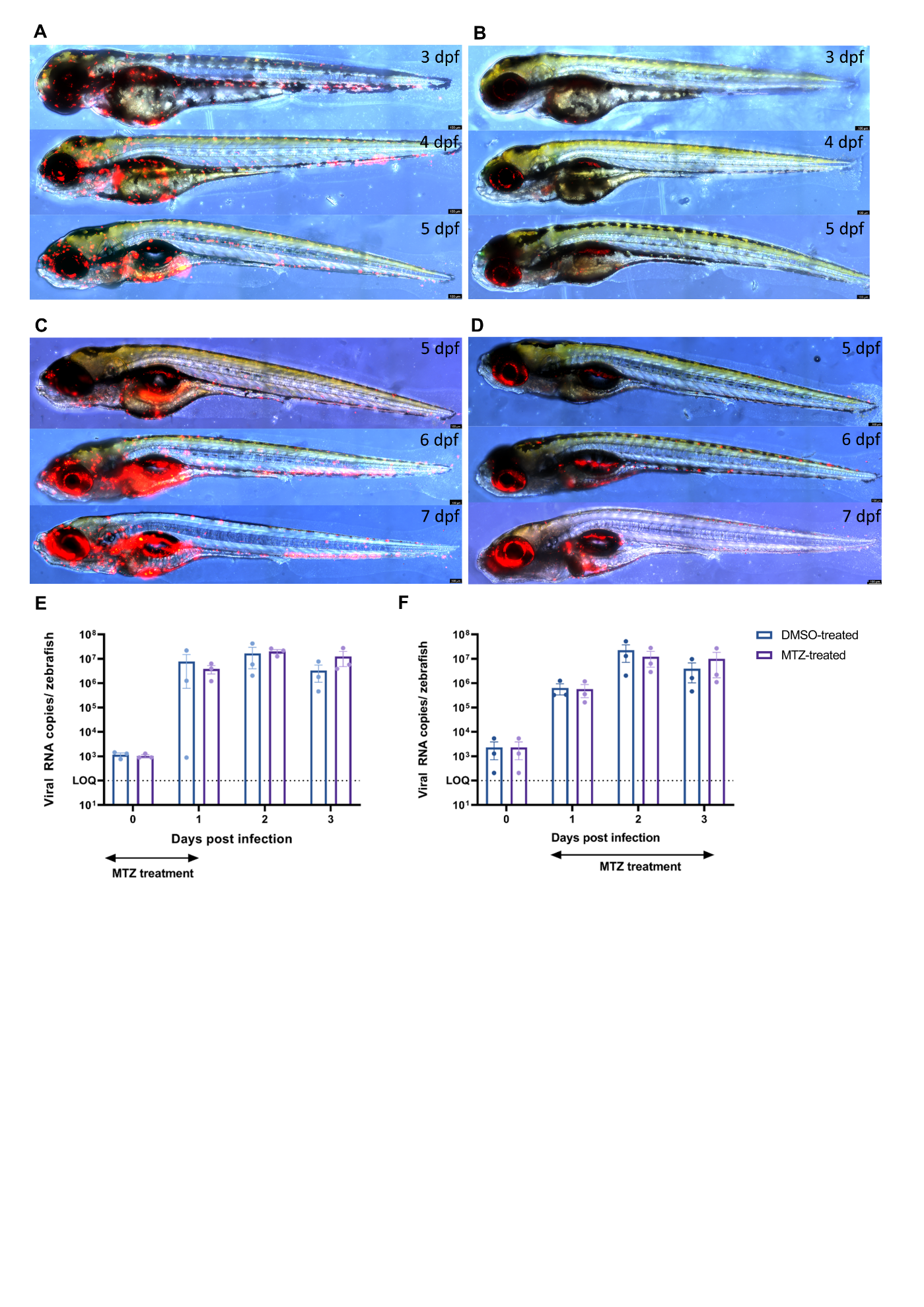

Supplement: Supplemental Material [file KGMI_A_2431167_SM7651.zip › Fig s7-s12.docx]

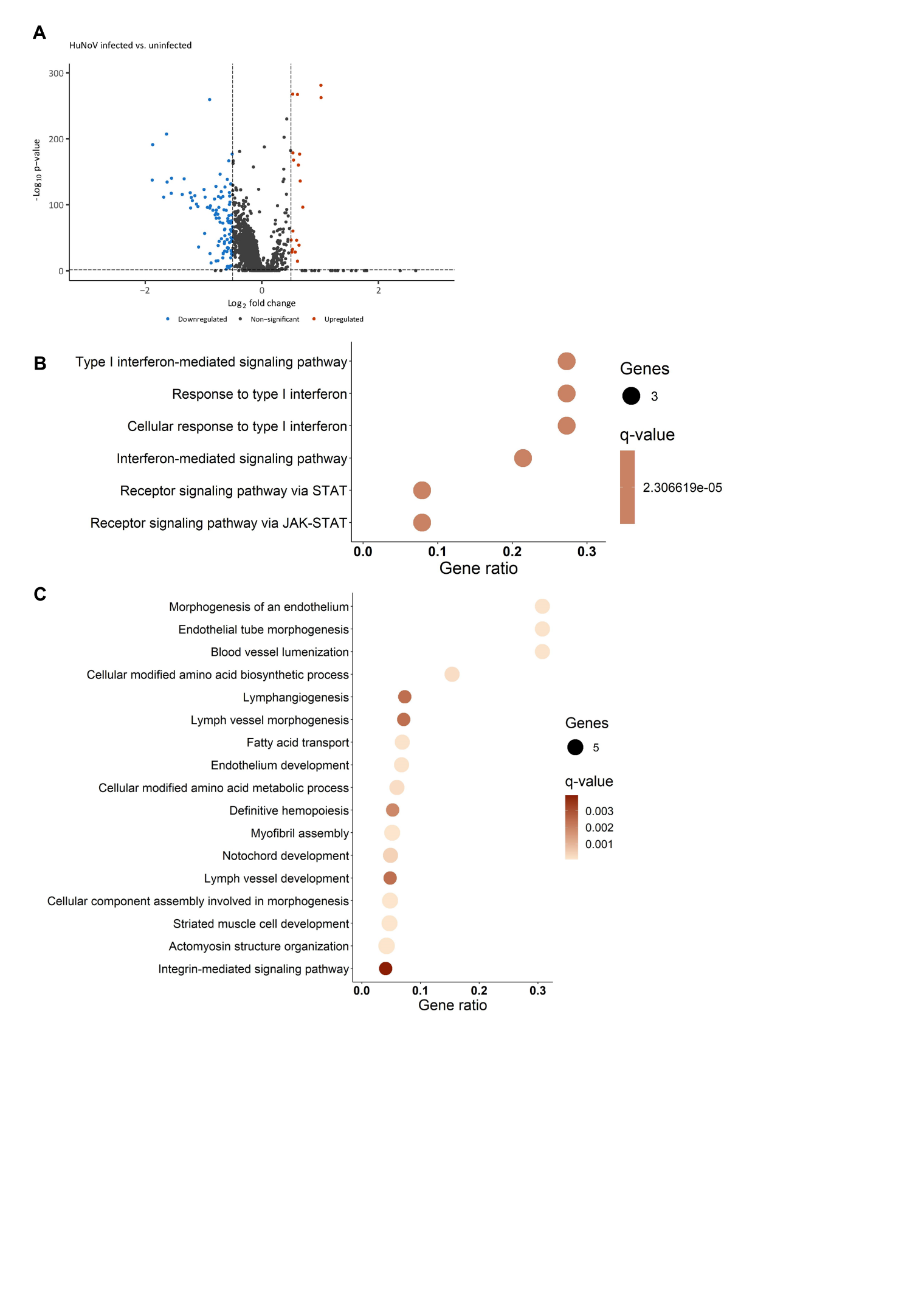
Fig s1

Fig s2


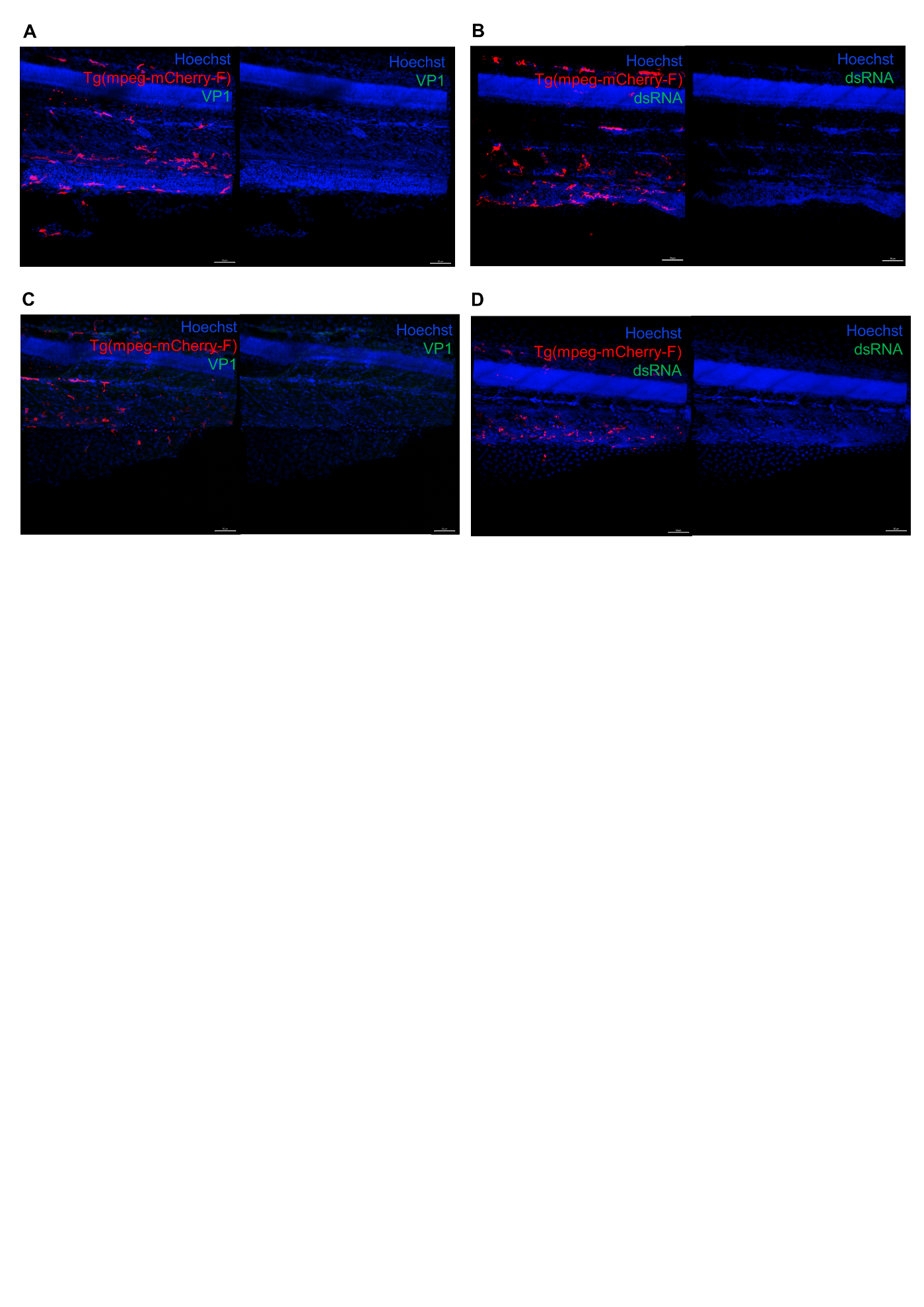


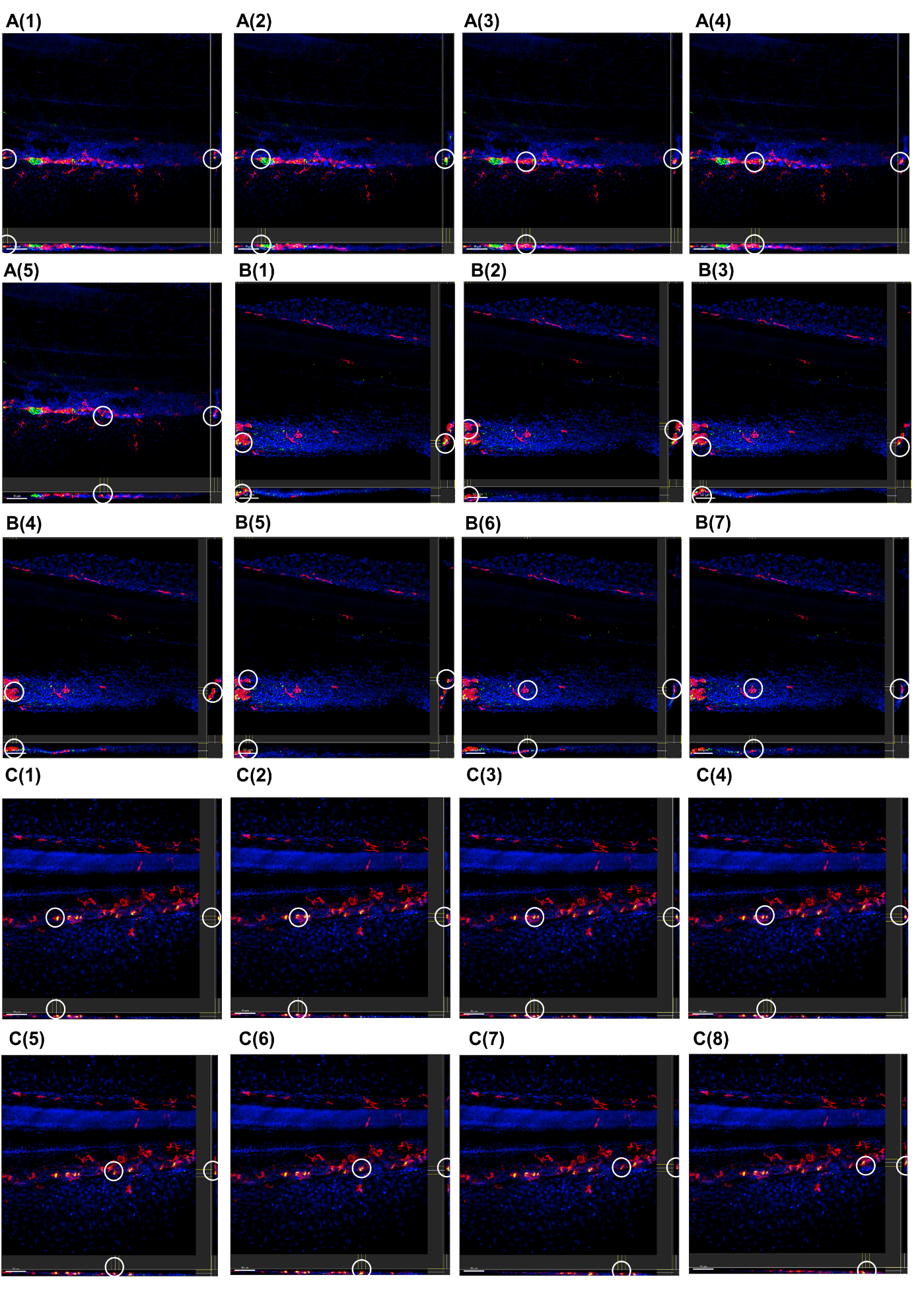
Fig s3

Fig s4


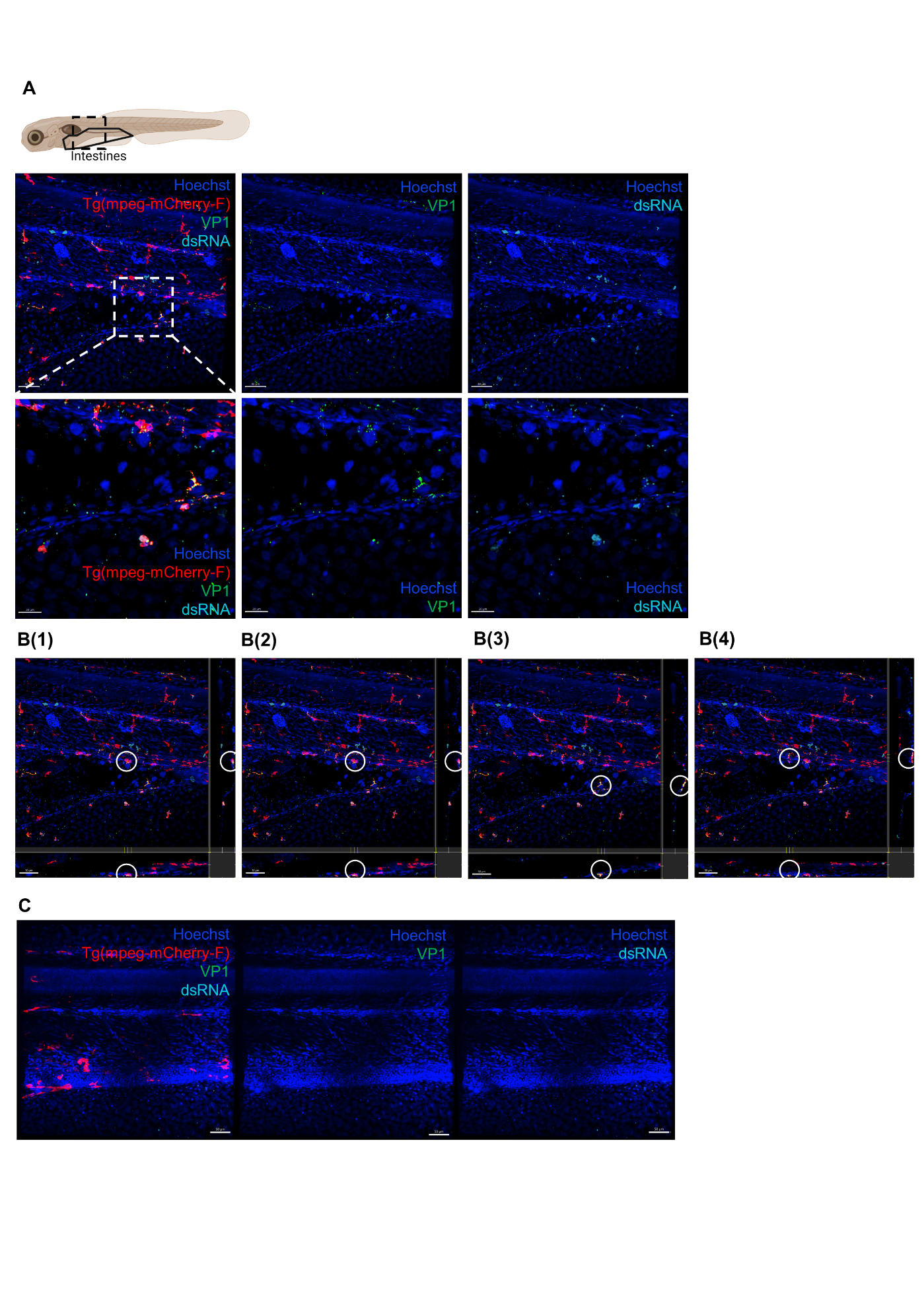


Fig s5


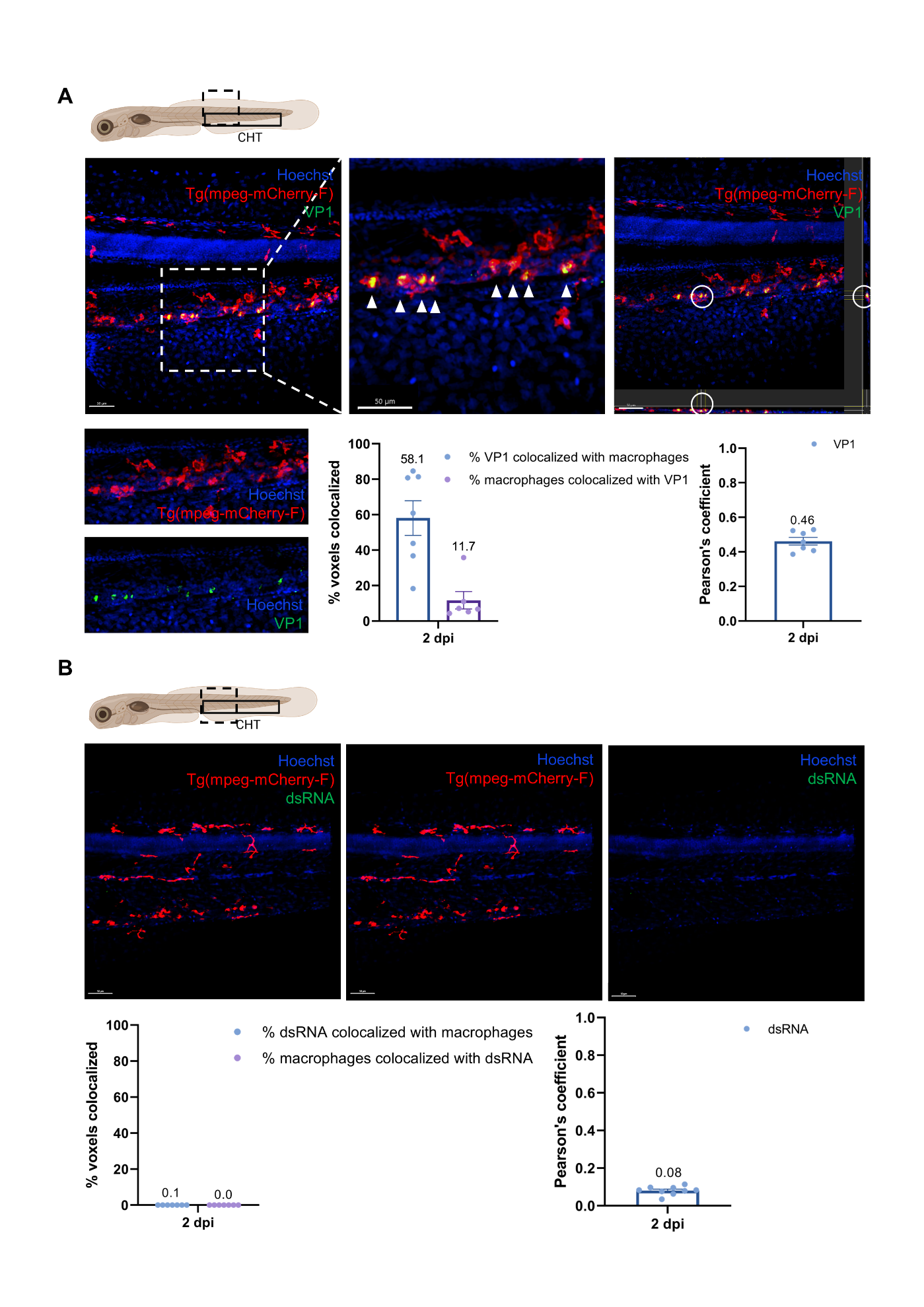


Fig s6


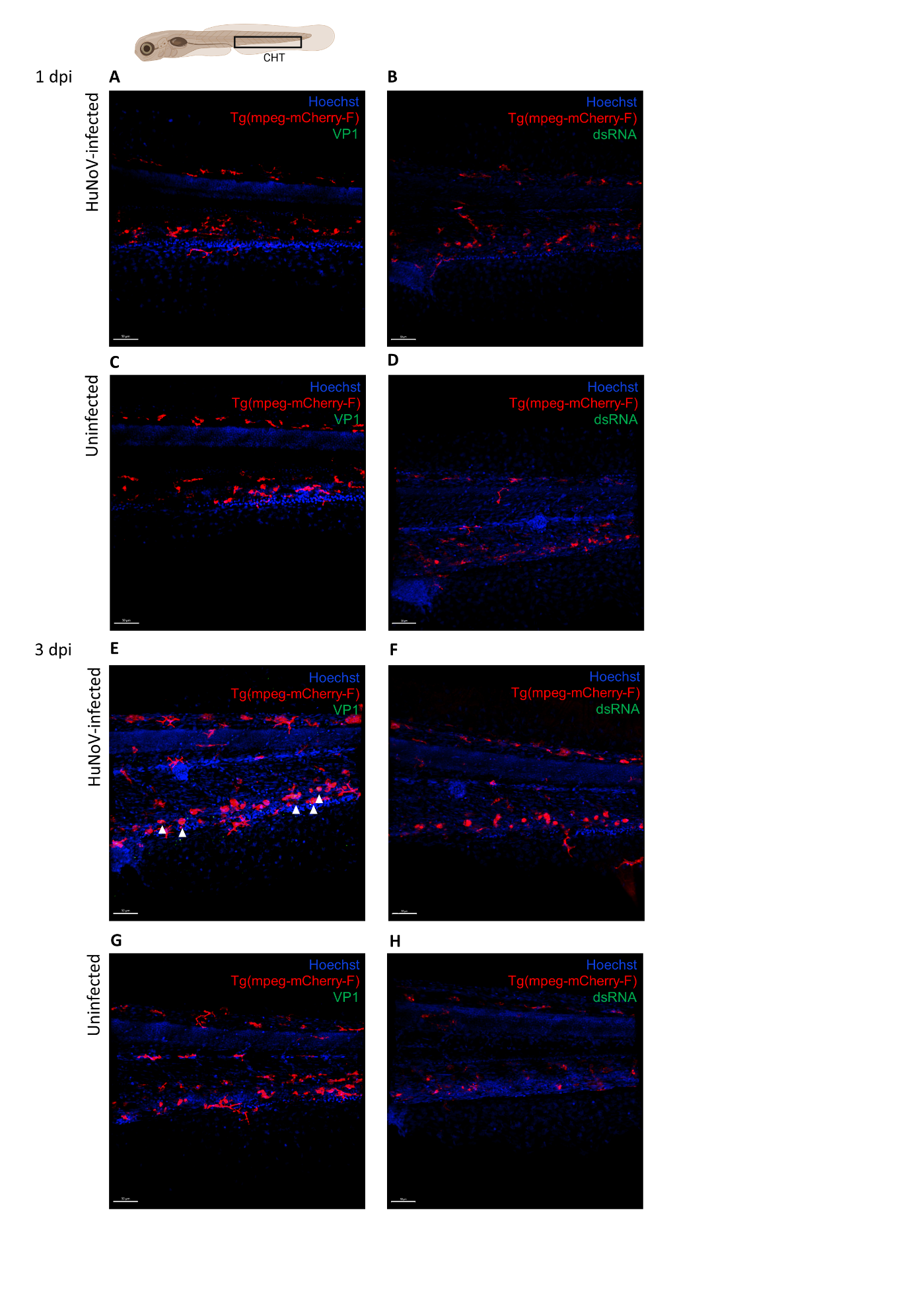

Supplement: Supplemental Material [file KGMI_A_2431167_SM7651.zip › fig s1-s6.docx]
